# Molecular Pathology of Sodium Channel Beta-Subunit Variants

**DOI:** 10.3389/fphar.2021.761275

**Published:** 2021-11-19

**Authors:** Paweorn Angsutararux, Wandi Zhu, Taylor L. Voelker, Jonathan R. Silva

**Affiliations:** ^1^ Department of Biomedical Engineering, Washington University in St. Louis, St. Louis, MO, United States; ^2^ Department of Medicine, Brigham and Women’s Hospital, Boston, MA, United States

**Keywords:** cardiac sodium channel, genetic variants, atrial fibrillation, brugada syndrome, beta-subunits

## Abstract

The voltage-gated Na^+^ channel regulates the initiation and propagation of the action potential in excitable cells. The major cardiac isoform Na_V_1.5, encoded by *SCN5A*, comprises a monomer with four homologous repeats (I-IV) that each contain a voltage sensing domain (VSD) and pore domain. In native myocytes, Na_V_1.5 forms a macromolecular complex with Na_V_β subunits and other regulatory proteins within the myocyte membrane to maintain normal cardiac function. Disturbance of the Na_V_ complex may manifest as deadly cardiac arrhythmias. Although *SCN5A* has long been identified as a gene associated with familial atrial fibrillation (AF) and Brugada Syndrome (BrS), other genetic contributors remain poorly understood. Emerging evidence suggests that mutations in the non-covalently interacting Na_V_β1 and Na_V_β3 are linked to both AF and BrS. Here, we investigated the molecular pathologies of 8 variants in Na_V_β1 and Na_V_β3. Our results reveal that Na_V_β1 and Na_V_β3 variants contribute to AF and BrS disease phenotypes by modulating both Na_V_1.5 expression and gating properties. Most AF-linked variants in the Na_V_β1 subunit do not alter the gating kinetics of the sodium channel, but rather modify the channel expression. In contrast, AF-related Na_V_β3 variants directly affect channel gating, altering voltage-dependent activation and the time course of recovery from inactivation via the modulation of VSD activation.

## Introduction

Electrical excitation of cardiomyocytes, the cardiac action potential, is initiated and propagated from myocyte to myocyte by the rapid conduction of inward Na^+^ current through voltage-gated Na^+^ (Na_V_) channels. Na_V_ channels in native myocytes comprise a macromolecular complex that contains the primary pore-forming α-subunit Na_V_1.5, and a myriad of accessory proteins that include the Na_V_ β-subunits, calmodulin and intracellular fibroblast growth factors (iFGFs) (Abriel and Kass, 2005; Abriel, 2010). The Na_V_ channel α-subunit is a pseudo-tetrameric protein, composed of four homologous repeats (I-IV). Each repeat contains six transmembrane segments (S1–S6), with the voltage sensing domain (VSD) formed by S1–S4 and the channel pore that is cooperatively defined by S5 and S6. The fourth segment (S4) carries multiple positively charged amino acids that activate by translocating across the membrane upon the change in membrane potential. VSD activation is coupled to pore dynamics, leading to a precise control of Na_V_ channel activation and inactivation kinetics. Na_V_ channel dysfunction caused by inherited variants can result in conduction diseases and deadly cardiac arrhythmias including atrial fibrillation (AF), long-QT syndrome (LQT), and Brugada syndrome (BrS) (Wang et al., 1995; Chen et al., 1998; Schott et al., 1999; Tan et al., 2001; Olson et al., 2005; Ellinor et al., 2008). Gain-of-function variants that increase persistent Na^+^ current by disrupting Na_V_ channel inactivation are diagnosed as LQT syndrome Type 3 (Wang et al., 1995). The opposite loss-of-function mutation resulting in less inward Na^+^ current is typically registered as conduction disease (Schott et al., 1999; Tan et al., 2001) and BrS (Chen et al., 1998), although there are several reports of overlapping phonotypes (Sandhu et al., 2017). Some mutations that alter Na_V_ channel gating properties and affect the duration of action potential are also found to predispose patients to AF (Olson et al., 2005; Ellinor et al., 2008).

The Na_V_ channel β-subunits have been previously shown to regulate Na_V_ channel function through multiple mechanisms including the modulation of expression at the myocyte membrane (Calhoun and Isom, 2014), altered kinetics (Fahmi et al., 2001; Watanabe et al., 2009; Calhoun and Isom, 2014), the mediation of cell adhesion (Isom et al., 1995; Malhotra et al., 2000; Yu et al., 2003) and the recruitment of cytosolic proteins (Winters and Isom, 2016). The co-expression of Na_V_ β-subunits also affects the channel’s pharmacological response (Lenkowski et al., 2003; Uebachs et al., 2010; Zhu et al., 2021). It is therefore unsurprising that an increasing number of genetic variants have been identified in Na_V_ β-subunits among patients with cardiac arrhythmias.

In humans, there are 5 isoforms of Na_V_ β-subunits (β1–β4 and β1b), encoded by *SCN1B-SCN4B* (Winters and Isom, 2016). All β1–β4 subunits share the same topology with an extracellular N-terminal immunoglobulin (Ig) domain, a transmembrane segment, and an intracellular C-terminus (Calhoun and Isom, 2014), except for β1b subunit that contains only the N-terminal domain (Patino et al., 2011). The β2 and β4 subunits interact with Na_V_ α-subunit through covalent disulfide bonds, whereas β1 and β3 subunits interact with α-subunit non-covalently (Isom et al., 1992; Morgan et al., 2000). In human heart, the most abundant isoforms are Na_V_1.5 and β1 subunit (Gaborit et al., 2007; Zhu et al., 2021), while other β isoforms are found to have distinct subcellular localization (Maier et al., 2004) with differential expression profile over embryonic development (Domίnguez et al., 2005; Okata et al., 2016). Recent studies show that β1 and β3 subunits employ different molecular mechanisms to regulate Na_V_ channel gating, leading to distinct Na_V_ channel kinetics and unique cell excitability profile (Zhu et al., 2017; Salvage et al., 2019; Zhu et al., 2019). Their distinct regulation mechanisms lead to differential Na_V_ channel pharmacological responses, contributing to chamber-specific drug profiles where they show a unique expression pattern of β1 and β3 subunits between atrial and ventricular cardiomyocytes (Fahmi et al., 2001; Watanabe et al., 2009; Yuan et al., 2014; Zhu et al., 2021).

Mutations in β-subunits were reported among patients with inherited arrhythmias including BrS (Watanabe et al., 2008; Hu et al., 2012), long-QT syndrome (Medeiros-Domingo et al., 2007; Riurό et al., 2014), sudden infant death syndrome (Hu et al., 2012; Calhoun and Isom, 2014) and AF (Watanabe et al., 2009; Olesen et al., 2011; Li et al., 2013). However, the underlying mechanism of how these variants cause different disease phenotypes are not well understood. Here, we systematically characterized 8 variants linked to AF or BrS on Na_V_ β1 and β3 subunits. We employed voltage-clamp fluorometry and biochemistry methods to dissect the underlying mechanisms related to each variant.

## Materials and Methods

### Molecular Biology

The cDNAs encoding the human Na_V_ β3 subunit (UniProtKB/Swiss-Prot accession no. Q07699.1) and human Na_V_ β1 (UniProtKB/Swiss-Prot accession no. Q9NY72) were inserted into the pBSTA plasmid. The cDNA for human Na_V_1.5 was produced from the pMAX vector. Point mutations were introduced via the QuikChange II site-directed mutagenesis kit (Agilent) with primers from Sigma-Aldrich. Colonies were picked and plasmids were isolated using the NucleoSpin plasmid miniprep kit (Macherey-Nagel) before sequencing (Genewiz). A single clone containing the designated mutation was then expanded with Midiprep preparation (NucleoBond Xtra Midi, Macherey-Nagel). Each plasmid was linearized with *Not*I or *Eco*RI restriction enzymes and purified with the NucleoSpin Gel and PCR Clean-up kit (Macherey-Nagel). The linearized DNA was then used to synthesize the capped mRNA with the mMessage mMachine T7 Transcription Kit (Life Technologies). The mRNA was finally purified via phenol-chloroform extraction and reconstituted to a concentration of ∼1 μg/μl.

### Cut-Open Oocyte Voltage-Clamp

The mRNAs for the human α-subunit Nav1.5 and β1 or β3 subunits were injected at a 1:3 M ratio (50–56 ng per cell total) into *Xenopus* oocytes. Oocytes were then incubated at 18°C in ND93 solution (mM: 93 NaCl, 5 KCl, 1.8 CaCl_2_, 1 MgCl_2_, 5 HEPES, and 2.5 Na-pyruvate, and 1% penicillin-streptomycin, pH 7.4). 3–7 days after injection, cut-open recordings (Siefani and Bezanilla, 1998; Rudokas et al., 2014) were performed using a cut-open amplifier (CA-1B, Dagan Corporation) coupled to an A/D converter (Digidata 1440; Molecular Devices). Clampex software (v10, Molecular Devices) was used for data acquisition. During records the temperature was maintained at 19°C with a controller (HCC-100A, Dagan Corporation). The internal recording solution was composed of (mM): 105 NMG-Mes, 10 NaMes, 20 HEPES, and 2 EGTA, pH 7.4, and the external recording solution was (mM): 25 NMG-Mes, 90 Na-Mes, 20 HEPES, and 2 Ca-Mes2, pH 7.4.

Prior to recording, membrane capacitance compensation and P/–8 leak subtraction were applied. Ionic currents were recorded using a standard current-voltage (I-V) protocol: from −100 mV holding potential, cells were stepped to a 100 ms pre-pulse of −120 mV, then stepped to test potentials ranging from −120 to 60 mV with 10 mV increment, followed by a 100 ms post-pulse of −120 mV. For steady-state inactivation, cells were held at the test potential for 200 ms, then availability was measured by a depolarizing pulse of −20 mV. For recovery from inactivation, cells were depolarized to −20 mV for 200 ms to induce inactivation, then after various recovery durations at −120 mV, ranging from 1–1000 ms, cells were tested at −20 mV to record the fraction of channels recovered and available to conduct current.

### Voltage Clamp Fluorometry

Before recording, oocytes were subjected to fluorescence labeling using methanethiosulfonate-carboxytetramethylrhodamine (MTS-TAMRA, Santa Cruz Biotechnology) at 10 μmol/L in a depolarizing solution (mM: 110 KCl, 1.5 MgCl2, 0.8 CaCl2, 0.2 EDTA and 10 HEPES, pH 7.1) for 30 min on ice. Fluorescence emission and ionic current were recorded simultaneously on a custom rig that combines cut-open voltage clamp and an epifluorescence upright microscope (FN1, Nikon) via a 40X water-immersion objective with 0.8 NA (CFI Plan Fluor, Nikon). A green, high-powered LED (Luminus, PT-121) provided the excitation source and was controlled through a driver (Lumina Power, LDPC-20-6-24VDC) by Clampex software. The emitted fluorescence signal was detected by a photodiode (PIN-040A, United Detector Technology) which then was amplified by a patch clamp amplifier (Axopatch-200A, Molecular Devices). The recording was repeated about 7–10 times for each cell to average the fluorescence traces recorded. The internal solution was (mM): 105 NMG-Mes, 10 Na-Mes, 20 HEPES, and 2 EGTA, pH 7.4, and the external solution contained (mM): 25 NMG-Mes, 90 Na-Mes, 20 HEPES, and 2 Ca-Mes_2_, pH7.4.

### Immunofluorescence

Oocytes were fixed using a protocol adapted from Gagnon and Mowry (2011). Four days after RNA injection, Na^+^ currents were recorded from *Xenopus* oocytes using cut-open voltage clamp to confirm normal channel expression. Subsequently, oocytes were treated with proteinase K solution (0.1M Tris-HCL pH 7.5, 10 mM EDTA, 50 μg/ml Proteinase K) for 3 min and fixed by shaking for 1 h in 4% formaldehyde in MEM solution (0.1 M MOPS, 2 mM EGTA, 1 mM MgSO_4_). After fixation, oocytes were washed 3 times, each time for 15 min, by rocking in PBT solution (1X PBS, 0.2% BSA, 0.1% Triton) at room temperature. The oocytes were then transferred to blocking solution (1X PBT, 2% BSA, 2% goat serum) and rocked for 2 h at room temperature. After blocking, oocytes were incubated with a mouse monoclonal Pan-Na_V_ antibody (S8809, Sigma), diluted 1:200 in blocking solution overnight at 4°C. On the following day, oocytes were again washed 3 times, each time for 30 min in PBT solution, and subsequently incubated with an Alexa 488-tagged goat-anti-mouse secondary antibody (A-11001, Invitrogen), diluted 1:200 in blocking solution for 1 h at room temperature. Oocytes were then washed 3 times, each time for 30 min in PBT solution. For oocytes expressing the myc-tagged β1, 1:400 rabbit anti-myc antibody (PA1-981, Thermo Fisher), diluted 1:400 in blocking solution, was used (4°C overnight incubation). Oocytes expressing Na_V_β3 were incubated in the rabbit anti-Na_V_ β3 antibody (Wong et al., 2005) at 1:300 dilution in blocking solution at 4°C overnight. After washing 3 times, at 30 min each in PBT, oocytes were incubated with an Alexa 633-tagged goat-anti-rabbit secondary antibody (A-21070, Invitrogen) diluted 1:500 in blocking solution for 1 h at room temperature. Following additional 3 washes, oocytes were dehydrated and stored in methanol. For imaging, oocytes were mounted and pressed on microscope slides. Mounted slides were then imaged with a confocal microscope (Zeiss LM700) with a 63X oil immersion objective. The green channel was illuminated with 488 nm laser, while the red channel was illuminated with 633 nm laser. Merged images were generated by overlaying the two channels using ImageJ.

### Data Analysis

The fluorescence traces were analyzed with Clampfit (v11, Molecular Devices). The signals were low-pass filtered at 1 kHz and then corrected for photo-bleaching by baseline subtraction, using the fluorescence trace obtained at −120 mV which is the resting potential as a model to calculate traces with no change in fluorescence at other potentials. The voltage-dependent activation (G-V) and steady-state inactivation (SSI) curves were fitted to a Boltzmann function (y = 1/[1 + exp ((V-V_1/2_)/k)]). The channel recovery from inactivation curves were fitted to bi-exponential functions, accounting for both fast and slow recovery time constants. The current traces at −20 mV were fitted for activation and inactivation kinetics by an exponential and bi-exponential function accordingly. The raw fluorescence traces after 50 ms were exponential fitted for the deactivation time constants. The error bars represented the standard errors of mean (S.E.M). One-way analysis of Variance (ANOVA) followed by Dunnett’s post-hoc test was used to compare values of multiple variants to the control wild type. The independent *t*-test was used for other statistical comparisons.

## Results

### Mapping of Cardiac Arrhythmogenic Variants on Na_V_ β-Subunits

**FIGURE 1:**
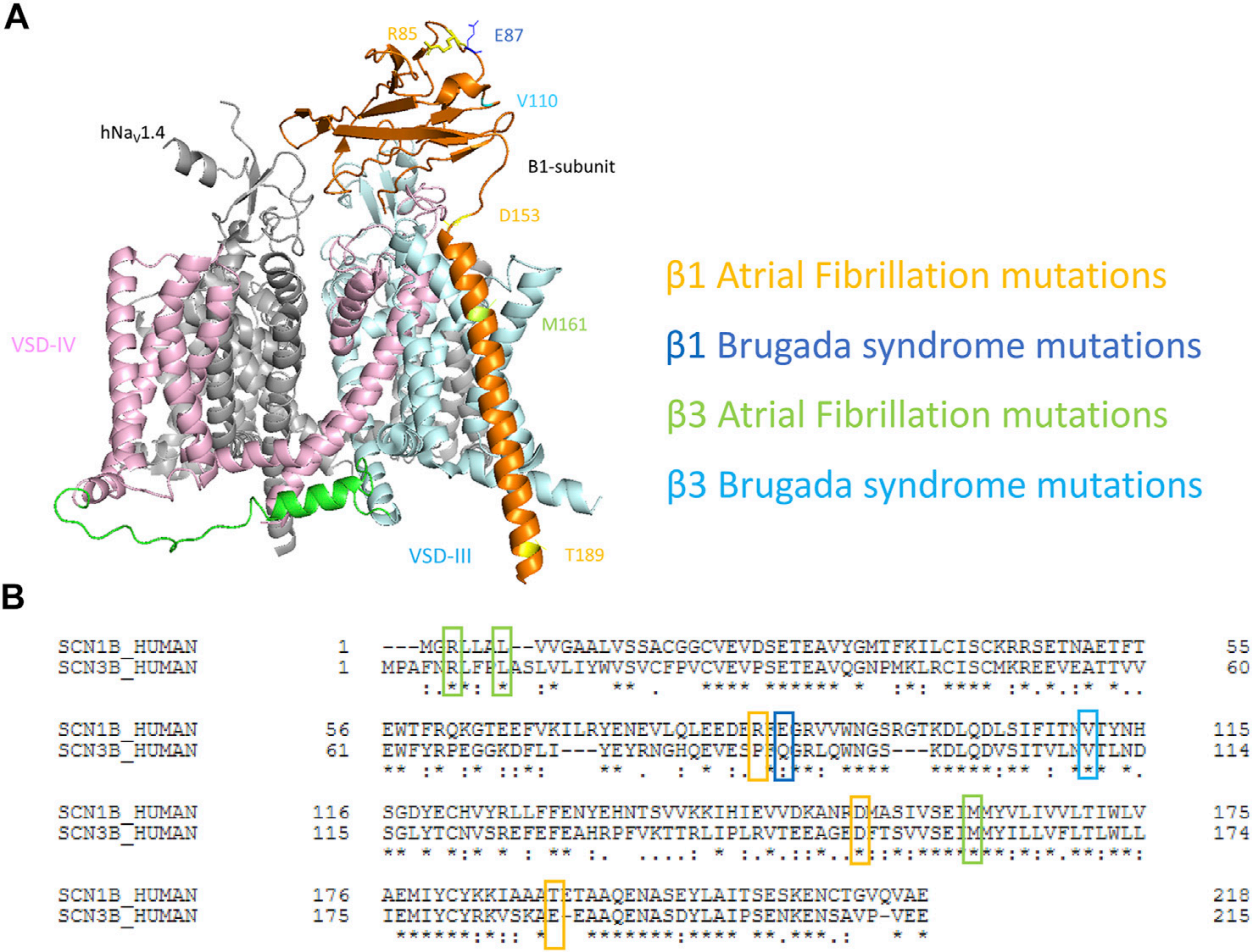
Mapping of cardiac disease-linked variants in β1 or β3 subunits onto a structure of hNaV1.4-β1 complex. **(A)** A structure of human muscle sodium channel and β1-subunit (hNa_V_1.4- β1 complex) (PDB: 6AGF) shown with the point mutations related to atrial fibrillation (AF) and Brugada syndrome (BrS) on either β1 or β3 subunits. **(B)** The alignment of human β1 and β3 subunits marked with cardiac disease-linked variants in this study.

Recently, the cryo-EM structures of multiple isoforms of human Na_V_ channels have been resolved as a complex between α- and β-subunits, including hNa_V_1.1-β1, hNa_V_1.2-β2, hNa_V_1.4-β1, and hNa_V_1.7-β1-β2 (Pan et al., 2018; Pan et al., 2019; Shen et al., 2019; Li et al., 2021a; Li et al., 2021b; Pan et al., 2021). The structures are highly similar among different human homologs. In human Na_V_1.4-β1 structures (PDB: 6AGF), β1 is tethered near VSD-III. Its transmembrane segment appears to interact with the VSD-III, while its Ig domain docks onto the surface constituting the extracellular loop of IV S6 and III S1-S2 (Figure 1A). Both the β1 and β3 subunits are known to express in human heart, interact non-covalently with Na_V_ α-subunit and show high similarities in their sequences (Morgan et al., 2000; Brackenbury and Isom, 2011) (Figure 1B). The structures of cardiac Na_V_1.5, both human and mouse, are also resolved, and superimpose well with the structures of other human isoforms (RMSD of Cα ∼0.7–1.4 A), except for the missing β1-subunit even when it is co-expressed, potentially due to the weak binding between β1 and Na_V_1.5 channel compared to other Na_V_ isoforms (Jiang et al., 2020; Angsutararux et al., 2021).

To understand the mechanisms of dysregulation connected to diseased-linked variants, we mapped all 8 arrhythmogenic variants on both β1 and β3 (β1: R85H, E87Q, D153N, T189M, and β3: R6K, L10P, V110I, M161T) in this study onto the structures of β1-hNa_V_1.4 complex. These variants are spatially located across the β-subunits, encompassing the Ig loop, the transmembrane segment, and the intracellular domain (Figure 1A). It should be noted that the structure of β1 from hNaV1.4- β1 only resolved residues 20–192. Thus, two BrS-linked variants (β3s R6K and L10P) were not mapped onto the structures. We aligned the sequences of β1 and β3 subunits (Figure 1B) and identified the β3 variants on the β1 structure accordingly. Most variants occur at the residues that are well conserved between mammalian Na_V_β1 and Na_V_β3. Most variants are located within the extracellular domain which constitutes an extensive portion of the β-subunit and plays an important role in the regulation of Na_V_ channel expression and modulation of channel gating (Makita et al., 1996; McCormick et al., 1998; McCormick et al., 1999; Yu et al., 2005). These Ig domain variants, however, are not located in the vicinity of interacting interface with α-subunit. Rather, they are associated with β-sheet folding, implying a role in determining the integrity of the Ig structure. The D153N variant in β1 constitutes the linker between the Ig and the transmembrane domain, where it can influence the orientation of Ig loop relative to the transmembrane segment (Glass et al., 2020). The transmembrane variant M161T is found within β3 where its transmembrane domain is engaged in the binding to α-subunit (Salvage et al., 2019). Lastly, T189M in β1 is found within the intracellular domain of β1-subunit that is required for its association with the α-subunit (Meadows et al., 2001; Zhu et al., 2017).

### Differential Effects of AF-Linked β1 and β3 Variants on Na_V_1.5 Channel Gating

**FIGURE 2:**
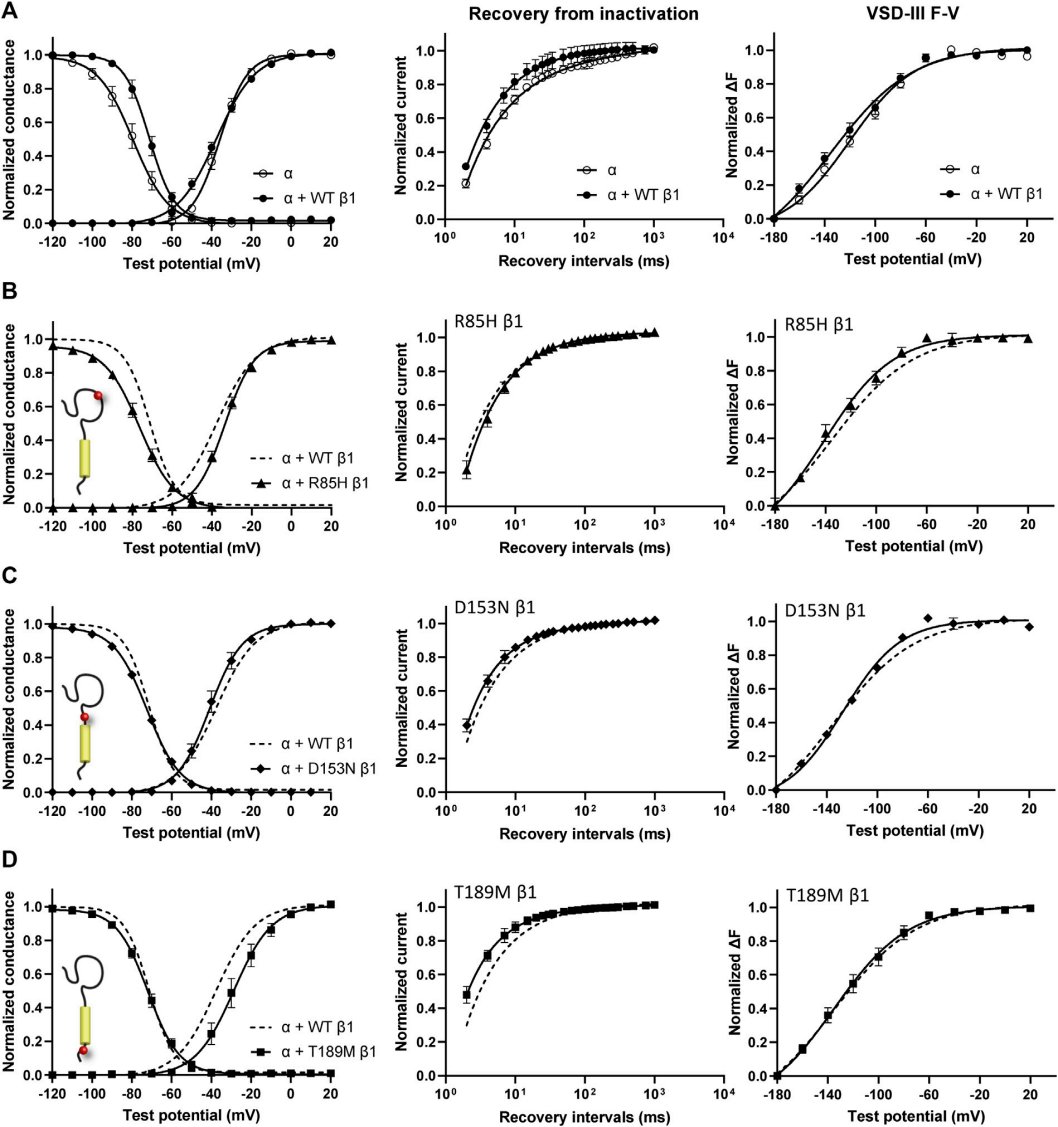
AF-linked β1 variants show diverse effects on NaV channel activation and inactivation curves, when compared to WT β1. Groups of 3–5 cells are reported as mean ± SEM. Data are fit with a Boltzmann equation. **(A)** Left: Voltage dependence of channel activation (G-V) and steady-state inactivation (SSI) curves of Na_V_1.5 alone (opened circle) and with WT β1 co-expression (filled circle). Middle: Time dependent Na_V_ channel recovery from inactivation of Na_V_1.5 alone and with WT β1 co-expression. The channel recovery is recorded as the ratio of channel available post 200 ms-conditioning pulse at −20 mV after varying recovery intervals of −120 mV. Right: Voltage dependence of steady-state fluorescence (F-V) curves of VSD-III LFS alone and with WT β1 co-expression. **(B)** The G-V and SSI curves **(left)**, recovery from inactivation (middle) and VSD-III F-V curve **(right)** of Na_V_1.5 co-expressed with R85H β1 (▲), in comparison to WT β1 (dashed line). **(C)** The G-V and SSI curves **(left)**, recovery from inactivation (middle) and VSD-III F-V curve **(right)** of Na_V_1.5 co-expressed with D153N β1 (♦), in comparison to WT β1 (dashed line). **(D)** The G-V and SSI curves **(left)**, recovery from inactivation (middle) and VSD-III F-V curve **(right)** of Na_V_1.5 co-expressed with T189M β1 (■), in comparison to WT β1 (dashed line).

Screening in lone AF patients identified three associated variants in β1, R85H and D153N (Watanabe et al., 2009) and T189M (Hayashi et al., 2015). A separate study also found β1 T189M in patients with sudden unexplained nocturnal death syndrome (SUNDS), or the death of healthy young males at night during sleep, a condition prevalent in the Southeast Asia (Liu et al., 2014). We co-expressed β1 containing these variants with human Na_V_1.5 in *Xenopus* oocytes at a molar ratio of 3:1 and examined their effect on Na_V_ channel gating, relative to the WT β1-subunit. The overexpressed ratio of β- to α-subunits is to ensure the saturation behavior of the β-subunit on the modulation effects of Na_V_1.5, as identified in a past study (Zhu et al., 2017). Consistent with our previously reported findings, we found that the co-expression of WT β1 caused a depolarizing shift in channel steady-state inactivation (SSI) compared to α-subunit expressed alone (Figure 2A, left; Supplementary Table S1; Zhu et al., 2017). Here, we observed that relative to the WT β1, none of the three AF-linked variants caused a significant shift in the half-maximal voltages of SSI curves, but the slope factor (k) was altered by R85H (*p* = 0.001) (Figures 2B–D, left; Table 1). Only T189M β1 altered the conductance-voltage (G-V) curve relative to WT β1, by inducing a depolarizing shift (Figure 2D, left; Table 1). Two variants, D153N and T189M β1s were found to facilitate recovery from inactivation, while R85H β1 did not affect the channel inactivation recovery rate relative to WT (Figures 2A–D, middle; Table 2). Consistently, the charge-voltage (Q-V) graph showed distinct effects between these variants. There was no change in the Q-V graph when β1 bears D153N or T189M variants, in comparison to WT β1 (Supplementary Figures S1B,C; Supplementary Table S2). The Q-V curve for R85H β1, in contrast, falls between the curves of WT β1 and of Na_V_1.5 alone (Supplementary Figure S1A; Supplementary Table S2). This effect suggests that R85H variant potentially alters β1 regulation of the channel gating charge.

**TABLE 1:** Parameters of Boltzmann fit to G-V, SSI and F-V curves for Na_v_1.5 expressed with WT or mutant β1s and *p*-values computed from one-way ANOVA followed by Donnett’s post hoc test (*, **, and *** indicate *p*-values of less than 0.05, 0.01, and 0.001 accordingly).

		WT β1	R85H β1	D153N β1	T189M β1	E87Q β1
G-V						
	V_1/2_	−37.6 ± 0.7	−33.6 ± 1.2	−40.7 ± 2.4	−28.7 ± 3.0	−38.9 ± 2.6
	ΔV_1/2_		4.0	−3.1	8.9	−1.3
	*p*-value		0.55	0.69	0.027 (*)	0.98
	k [n]	8.8 ± 0.4 [5]	7.3 ± 0.1 [4]	7.8 ± 0.5 [5]	8.8 ± 0.8 [5]	6.7 ± 1.0 [4]
SSI						
	V_1/2_	−71.4 ± 0.3	−77.0 ± 1.1	−72.7 ± 0.2	−72.1 ± 1.3	−61.7 ± 2.0
	ΔV_1/2_		−5.6	−1.3	−0.7	9.7
	*p*-value		0.064	0.94	0.99	<0.001 (***)
	k [n]	−6.0 ± 0.1 [4]	−9.3 ± 0.3 [4]	−8.6 ± 0.5 [4]	−7.9 ± 0.2 [5]	4.6 ± 0.4 [5]
DIII F-V						
	V_1/2_	−134.6 ± 6.4	−137.8 ± 5.1	−133.8 ± 2.4	−133.0 ± 6.8	−144.3 ± 0.3
	ΔV_1/2_		−3.2	0.8	1.6	-9.7
	*p*-value		0.98	0.99	0.99	0.52
	k [n]	31.3 ± 0.5 [4]	25.6 ± 2.3 [4]	25.5 ± 2.6 [3]	27.6 ± 1.4 [4]	21.2 ± 1.0 [4]
DIV F-V						
	V_1/2_	−67.9 ± 3.1	−61.6 ± 1.3	−63.8 ± 0.4	−60.4 ± 1.5	−71.6 ± 2.6
	ΔV_1/2_		6.3	4.1	7.5	−3.8
	*p*-value		0.18	0.52	0.093	0.50
	k [n]	15.1 ± 0.4 [4]	16.4 ± 3.5 [4]	11.6 ± 2.0 [4]	11.6 ± 0.3 [4]	11.1 ± 0.9 [6]

**TABLE 2:** The recovery time constants (Ƭ_R_) were computed from the bi-exponential fit of the inactivation recovery curves for Na_v_1.5 co-expressed with WT or mutant β1s. The inactivation time constants (Ƭ_I_) and the half-activation times (Ƭ_1/2_) for Na_V_1.5 co-expressed with WT or mutant β3s were fitted bi-exponentially and exponentially to the sodium current traces at −20 mV. Results were statistically tested by one-way ANOVA followed by Dunnett’s post-hoc test.

		WT β1	R85H β1	D153N β1	T189M β1	E87Q β1
Recovery	Ƭ_R_ 1	48.6 ± 2.6	47.1 ± 6.4	30.7 ± 4.6	19.3 ± 2.3	11.7 ± 1.5
	*p*-value		0.99	0.035 (*)	<0.001 (***)	<0.001 (***)
	Ƭ_R_ 2	5.2 ± 0.4	4.7 ± 0.5	3.2 ± 0.5	2.4 ± 0.2	1.5 ± 0.2
	*p*-value		0.92	0.05 (*)	0.0052 (**)	<0.001 (***)
	N	3	3	3	3	5
		**WT β3**	**R6K β3**	**L10P β3**	**M161T β3**	
Inactivation						
	Ƭ_I_ 1	6.8 ± 0.3	10.2 ± 1.1	9.4 ± 0.3	7.7 ± 0.8	
	*p*-value		0.005 (**)	0.04 (*)	0.65	
	Ƭ_I_ 2	1.0 ± 0.1	1.9 ± 0.2	1.1 ± 0.1	1.0 ± 0.02	
	*p*-value		0.001 (***)	0.84	>0.99	
	N	6	4	3	3	
Activation						
	Ƭ_1/2_	0.29	0.50	0.37	0.34	
	*p*-value		<0.001 (***)	0.048 (*)	0.38	
	N	4	4	5	4	

**FIGURE 3:**
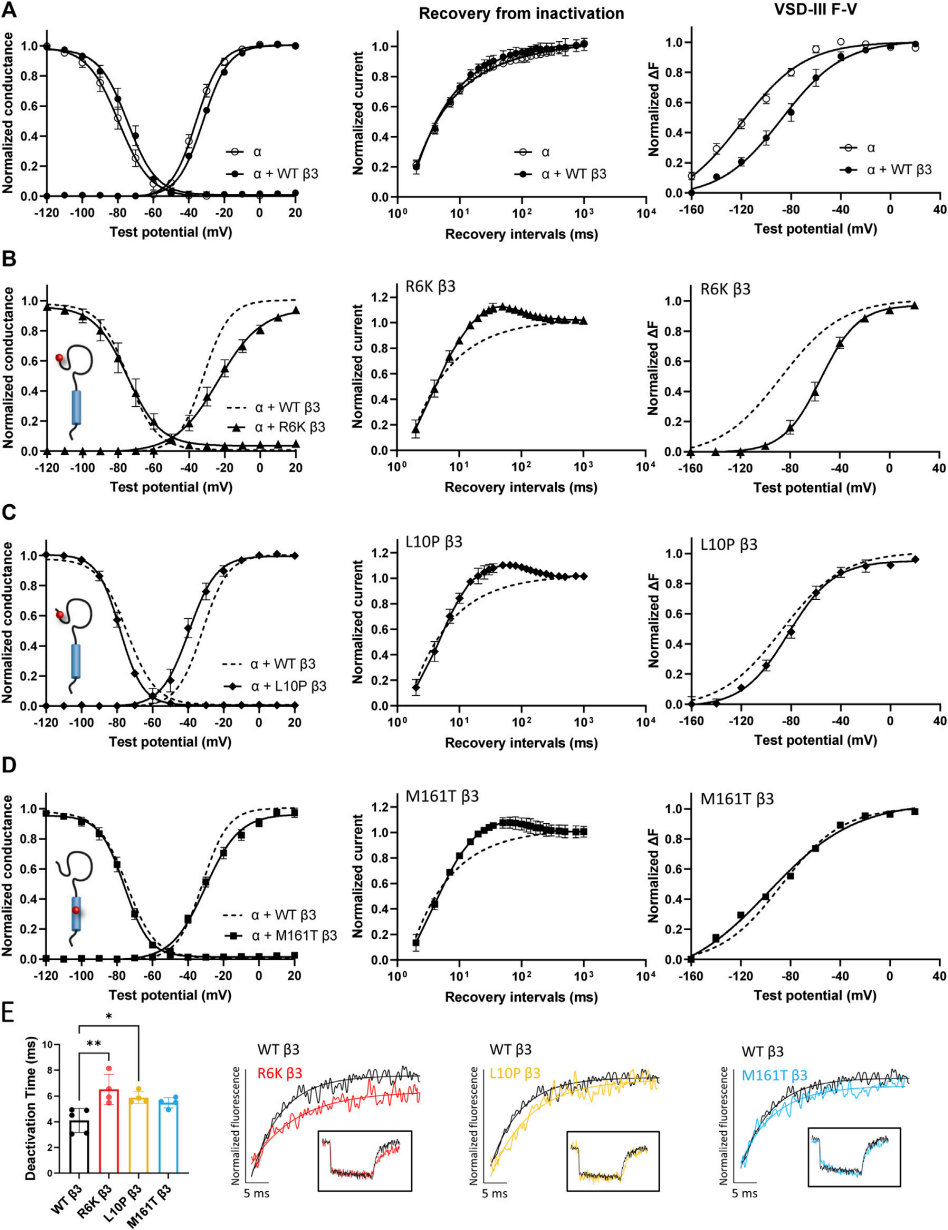
AF-linked β3 variants alter the NaV1.5 voltage dependence activation and VSD-III fluorescence emission curves relative to WT β3. Groups of 3-5 cells are reported as mean ± SEM. Data are fit with a Boltzmann equation. **(A)** Left: The G-V and SSI curves of Na_V_1.5 alone (opened circle) and with WT β3 co-expression (filled circle). Middle: Time dependent Na_V_ channel recovery from inactivation of Na_V_1.5 alone and with WT β3 co-expression. Right: Voltage dependence of steady-state fluorescence (F-V) curves of VSD-III VCF alone and with WT β3 co-expression. **(B)** The G-V and SSI curves **(left)**, recovery from inactivation (middle) and VSD-III F-V curve **(right)** of Na_V_1.5 co-expressed with R6K β3 (▲), in comparison to WT β3 (dashed line). **(C)** The G-V and SSI curves **(left)**, recovery from inactivation (middle) and VSD-III F-V curve **(right)** of Na_V_1.5 co-expressed with L10P β3 (♦), in comparison to WT β3 (dashed line). **(D)** The G-V and SSI curves **(left)**, recovery from inactivation (middle) and VSD-III F-V curve **(right)** of Na_V_1.5 co-expressed with M161T β3 (■), in comparison to WT β3 (dashed line). **(E)** A bar graph shows the VSD-III deactivation time constants exponential fitted to the fluorescence traces after 50 ms (* and ** indicate *p*-values of less than 0.05 and 0.01 accordingly). *p*-values calculated from one-way ANOVA followed by Dunnett’s post-hoc test are 0.002, 0.02, and 0.07 for R6K, L10P ,and M161T accordingly. Representative fluorescence traces during VSD-III deactivation obtained from VCF recordings at 20 mV of VSD-III LFS co-expressed with R6K (red), L10P (yellow) and M161T (blue) β3 variants relative to WT β3 (black).

Three β3 variants, R6K, L10P and M161T, were also identified in patients with lone AF (Olesen et al., 2011). The L10P β3 variant was additionally reported prior in a patient with BrS (Hu et al., 2009). The co-expression of WT β3 with α-subunit shifts the sodium channel steady-state inactivation curves towards depolarized potential (Figure 3A, left; Supplementary Table S1). We found that there was no modulatory effect from these AF-linked β3 variants on the channel availability curves in relative to WT β3 (Figures 3B–D, left). The channel activation curve, however, was shifted towards positive potentials by R6K, whereas L10P variant caused a hyperpolarizing shift compared to WT β3 (Figures 3B,C, left; Table 3). M161T did not significantly affect half-maximal activation potential but increased the slope factor (k) of the activation curve (*p* = 0.04) (Figure 3D, left; Table 3). In comparison to WT β3, two AF-linked β3 variants, R6K and L10P, showed slower current activation and inactivation kinetics as observed by the increased half activation time and inactivation time constants fit to the sodium current traces at −20 mV (Table 2; Supplementary Figure S3A). The Q-V curves for channels co-expressed with AF-linked β3 variants showed depolarizing shift compared to WT β3 co-expression (Supplementary Figures S1D–F; Supplementary Table S2), suggesting that the β3 variants enhance the regulation of VSD movement. Furthermore, the recovery from inactivation observed in all three AF variants is distinct from that of WT β3 co-expression but resembles the phenotype of overexpressed β3 (at 6 to 1 M ratio) (Zhu et al., 2017), with the number of available channels after a short recovery period (10–300 ms) exceeding channel availability following the initial control pulse (Figures 3A–D, middle).

**TABLE 3:** Parameters of Boltzmann fit to G-V, SSI and F-V curves for Na_v_1.5 expressed with WT or mutant β3s and *p*-values computed from one-way ANOVA followed by Donnett’s post hoc test.

		WT β3	R6K β3	L10P β3	M161T β3	V110I β3
G-V						
	V_1/2_	−31.9 ± 0.4	−22.6 ± 3.3	−42.1 ± 3.1	−28.9 ± 2.7	−35.4 ± 1.5
	ΔV_1/2_		9.3	−10.2	3.0	−3.5
	*p*-value		0.035 (*)	0.021 (*)	0.76	0.65
	k [n]	7.2 ± 0.2 [4]	12.6 ± 0.4 [4]	7.0 ± 0.9 [4]	9.4 ± 1.5 [4]	6.3 ± 0.8 [4]
SSI						
	V_1/2_	−72.4 ± 1.5	−75.9 ± 4.1	−78.4 ± 1.1	−76.5 ± 1.5	−70.2 ± 0.7
	ΔV_1/2_		−3.5	−6.0	−4.1	2.2
	*p*-value		0.42	0.081	0.30	0.71
	k [n]	−8.2 ± 0.3 [3]	−9.2 ± 0.7 [4]	−6.3 ± 0.3 [4]	−7.4 ± 0.7 [4]	−6.4 ± 0.5 [5]
DIII F-V						
	V_1/2_	−88.0 ± 3.2	−56.6 ± 3.5	−80.1 ± 5.9	−97.7 ± 3.4	−71.3 ± 0.4
	ΔV_1/2_		31.4	7.9	−9.7	16.7
	*p*-value		<0.001 (***)	0.31	0.17	0.0043 (**)
	k [n]	24.0 ± 2.0 [7]	16.6 ± 1.4 [4]	18.8 ± 1.5 [4]	34.3 ± 1.1 [4]	22.8 ± 1.3 [5]
DIV F-V						
	V_1/2_	−73.7 ± 1.4	−70.8 ± 7.0	−74.1 ± 4.0	−64.9 ± 1.5	−75.7 ± 1.6
	ΔV_1/2_		2.9	-0.4	8.8	−2.0
	*p*-value		0.95	0.99	0.33	0.98
	k [n]	20.5 ± 1.2 [4]	19.8 ± 3.0 [4]	15.2 ± 2.7 [4]	18.1 ± 1.8 [4]	20.6 ± 3.5 [4]

### Regulation of VSD-III Activation is Altered by AF-Linked Variants on β3, But Not on β1

The voltage clamp fluorometry (VCF) technique relies on a fluorescent molecule (MTS-TAMRA) to track the conformation of specific VSD in response to multiple voltage pulses and correlates its kinetics to the gating of ionic current. The Q-V curve can be reconstructed from the valence-weighted average midpoint of all VSD voltage-dependence fluorescence (F-V) curves (Varga et al., 2015). VCF thus provides valuable insights on the regulatory mechanisms of ion channels (Cha et al., 1999; Chanda and Bezanilla, 2002). Previous investigation on the mechanism of non-covalent β-subunits on the regulation of hNa_V_1.5 identified two important VSDs. Na_V_β1 caused a depolarizing shift in VSD-IV F-V curve, that estimates the change in VSD conformation (Figure 2A, right; Supplementary Figures S2A,B; Supplementary Table S1; Zhu et al., 2017), whereas Na_V_β3 altered both VSD-III and VSD-IV activation (Figure 3A, right; Supplementary Figures S3B,C; Supplementary Table S1; Zhu et al., 2017). We employed the same VCF constructs to examine the AF variants effect on VSD-III (Na_V_1.5 M1296C) and VSD-IV (Na_V_1.5 S1618C) (Supplementary Figures S2D,E; Varga et al., 2015). The co-expressions of AF-linked β1 variants did not cause any significant difference in the F-V curves of VSD-IV when compared to WT β1 (Table 1; Supplementary Figure S2C). We observed no shift in the half-activation voltage by β1 variants in VSD-III F-V curves relative to WT β1, but an altered slope factor (k) by R85H (*p* = 0.03) (Figures 2B–D, right; Table 1). This steeper slope results in more activated VSD-III in the higher voltage range, agreeing with the finding from the Q-V curve that R85H causes a hyperpolarizing shift in the gating charge movement. Overall, AF variants on β1 do not drastically affect the β1’s effect on Na_V_ channel α-subunit gating.

The measurement of VSD-III in the presence of AF-linked β3 variants, however, revealed a depolarizing shift in the F-V curves caused by the extracellular variant, R6K (Figure 3B, right; Table 3; Supplementary Figure S3E, left). The other extracellular variant L10P caused a depolarizing shift at negative potentials without significantly altering the F-V V_1/2_ (Figure 3C, right; Supplementary Figure S3E, middle). This additional depolarizing shift compared to WT β3 suggests that these two extracellular AF-related variants accentuate the β3 interaction with VSD-III. The M161T variant decreased the slope of the F-V curve (*p* = 0.01) (Figure 3D, right; Supplementary Figure S3E, right), suggesting an alteration in the number of charge or the trajectory of charge movement upon VSD-III activation. The depolarizing shifts in VSD-III activation from R6K and L10P β3 variants were consistent with slowed current activation kinetics (Supplementary Figure S3A) when VSD-III activation becomes rate-limiting step for activation gate opening. The half activation times of currents at −20 mV were prolonged by these two variants relative to WT β3 (Table 2). The raw fluorescence traces also show the slowed VSD-III deactivation of R6K and L10P β3 variants, as quantified by the increased deactivation time constants exponential fitted to the fluorescence traces, in comparison to the WT β3 (Figure 3E). The return of VSD-III upon the end of depolarization determines the channel recovery from inactivation as VSD-III and VSD-IV are immobilized by the inactivation gate during fast inactivation in Na_V_1.5 (Hsu et al., 2017). The slowed return of VSD-III by β3 variants can facilitate channel opening and leads to increased ionic current over 10–300 ms recovery duration. The activation of VSD-IV was not affected by any of the AF-linked β3 variants (Table 3; Supplementary Figures S3D,F).

### Altered Na_V_1.5 Expression by AF-Related β1 Variants, But Not AF-Related β3 Variants

**FIGURE 4:**
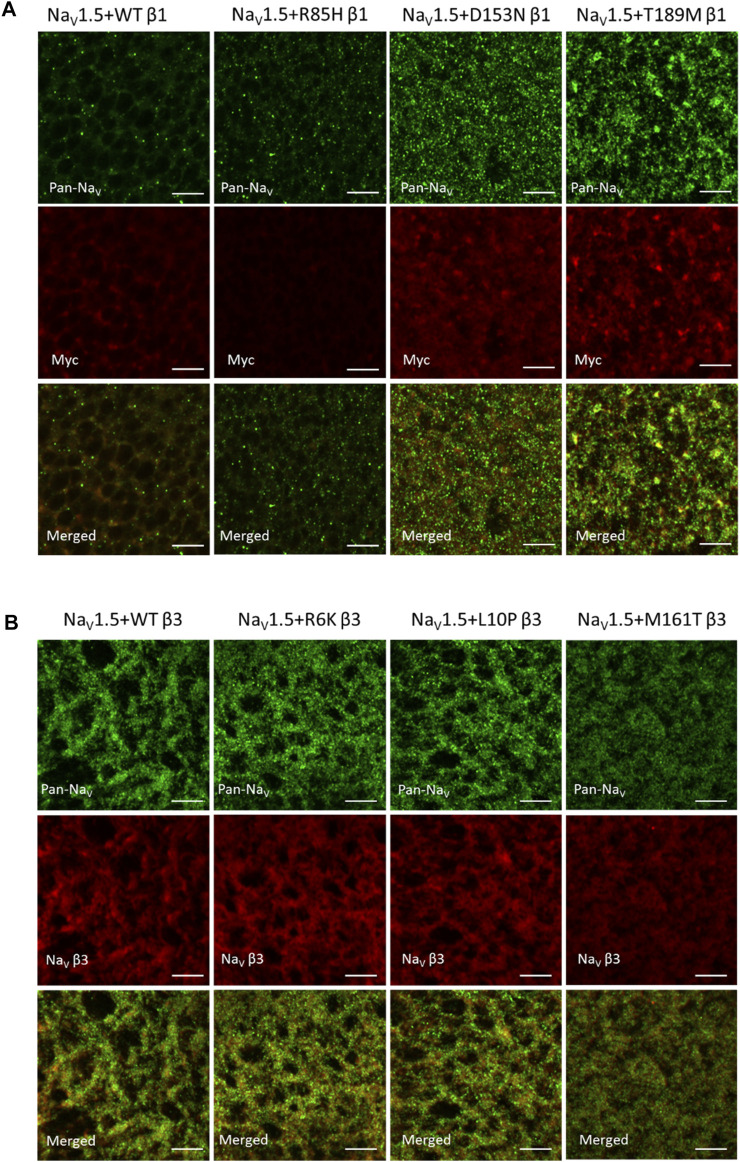
AF-related β1, but not β3 variants affect the NaV channel surface expression, as illustrated by immuno-fluorescence staining of NaV1.5 α- and β-subunits. 4 days after injection, *Xenopus* oocytes were fixed, stained and imaged with confocal microscope. Scale bars represent 5 µm. **(A)** *Xenopus* oocytes co-injected with Na_V_1.5 and WT, R85H, D153N, or T189M β1-cmycs were immuno-stained with mouse anti-PanNa_V_ antibody and rabbit anti-myc antibody. The surface expression of Na_V_1.5 and β1 were labeled in green and red accordingly. The last row showed the combined images of Na_V_1.5 and β1 labeling. **(B)** *Xenopus* oocytes co-injected with Na_V_1.5 and WT, R6K, L10P or M161T β3s were immuno-stained with mouse anti-PanNa_V_ antibody and rabbit anti-scn3b antibody. The surface expression of Na_V_1.5 and β3 were labeled in green and red accordingly. The last row showed the combined images of Na_V_1.5 and β3 labeling.

Na_V_ β-subunits were previously found to promote the cell surface expression of Na_V_ channel complex, resulting in an increase in current density (Calhoun and Isom, 2014). Na_V_ α-subunit also assembles into cluster on the plasma membrane, whose sizes and distances between neighboring clusters are regulated by β3-subunit (Salvage et al., 2020). We examined the channel expression of Na_V_1.5 α- and β-subunits on the surface membrane of *Xenopus* oocytes to study the effects of AF variants on Na_V_ channel trafficking. A myc-tag was added to the C-terminus of all β1-subunits since none of the commercially available *SCN1B* antibodies yield detectable signal (Cell Signaling Technology 14684S, Abcam ab107370). The additional myc-tag does not affect the gating regulation of the Na_V_1.5 (data not shown). Through immunostaining, both D153N and T189M variants increased the surface expression of Na_V_ α- and β-subunits, while the R85H variant showed the opposite effect (Mean fluorescence intensity/pixel (*n* = 3): WT β1 = 30.8 ± 2.4, R85H β1 = 13.7 ± 0.3, D153N β1 = 33.2 ± 2.6, T189M β1 = 43.1 ± 4.3) (Figure 4A). Comparison illustrates the parallel expression between Na_V_ α- and β-subunits, except for R85H variant where only β-subunit surface expression is affected but not Na_V_1.5. We also observed the larger puncta in T189M variant where α- and β-subunits assemble. The merged image of D153N variant shows the colocalization of α- and β-subunit, but the spots remain small.

Unlike AF-linked β1 variants, the AF-linked β3 variants, R6K, L10P, and M161T, did not alter Na_V_1.5 or β3-subunits expression level, compared to the WT β3 (Figure 4B). There was an overall increase in expression of Na_V_1.5 when β3 subunits are co-expressed, compared to the β1 subunits. In un-injected oocytes, we observed minimal staining by the Pan-Na_V_, Myc, and Na_V_ β3 antibodies, suggesting the immunofluorescence signals observed are specific for the Na_V_, β1 and β3 proteins (Supplementary Figure S4).

### BrS-Linked Variants on β1 and β3 Subunits Show Different Underlying Mechanisms

**FIGURE 5:**
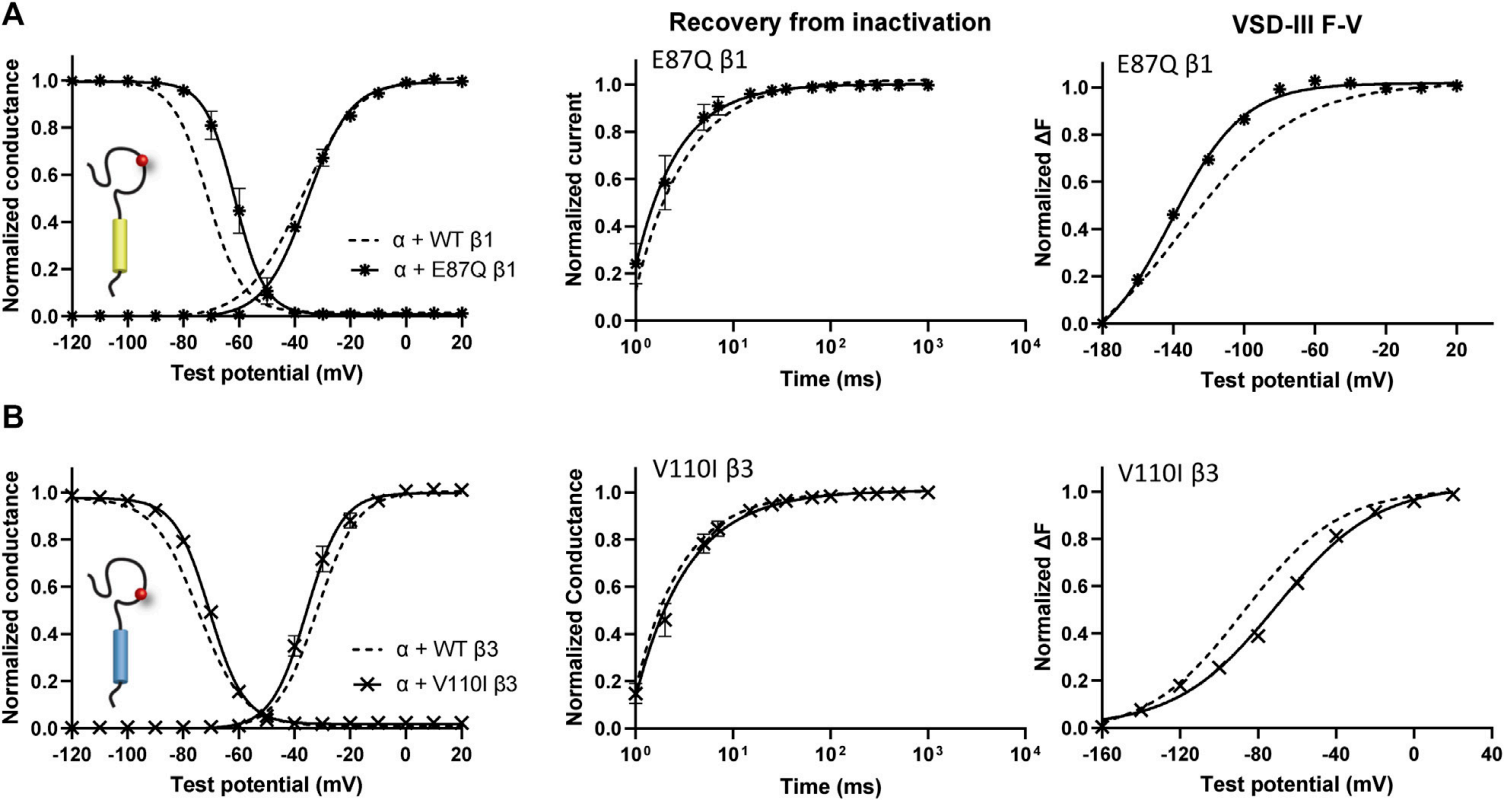
BrS-linked β1 variant alters the NaV channel steady-state inactivation, and VSD-III activation relative to WT β1. Groups of 3–5 cells are reported as mean ± SEM. Data are fit with a Boltzmann equation. **(A)** The G-V and SSI curves **(left)**, recovery from inactivation (middle) and VSD-III F-V curve **(right)** of Na_V_1.5 co-expressed with E87Q β1 (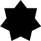), compared to WT β1 (dashed line). **(B)** The G-V and SSI curves **(left)**, recovery from inactivation (middle) and VSD-III F-V curve **(right)** of Na_V_1.5 co-expressed with V110I β3 (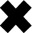), in comparison to WT β3 (dashed line).

**FIGURE 6:**
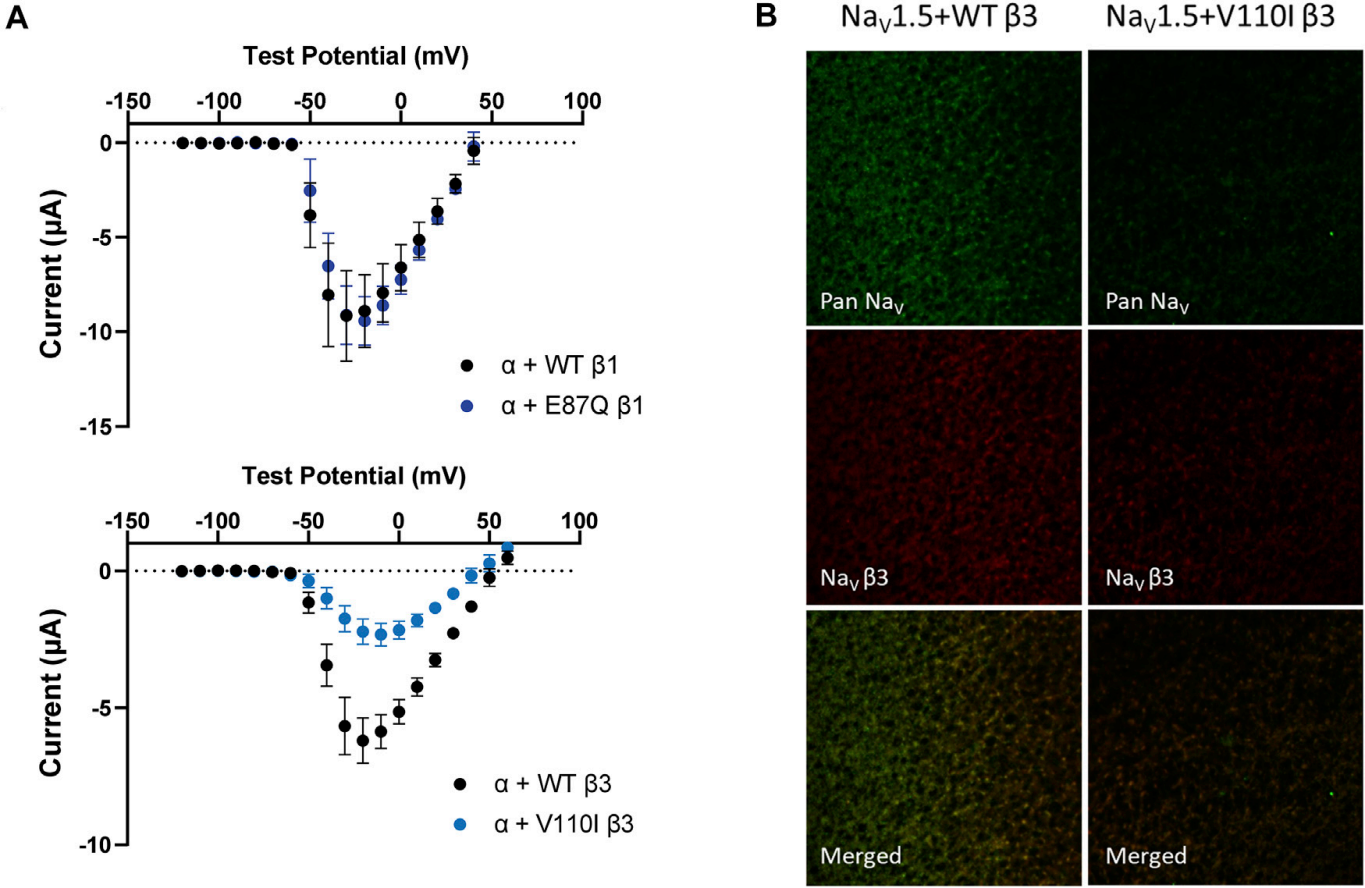
BrS-linked β3 variant reduces the peak current amplitude and the cell membrane expression of NaV channel complex, while BrS-linked β1 variant does not. Current is recorded by cut-open voltage clamp with the same cell area. Groups of 3–5 cells are report as mean ± SEM. Immunofluorescence staining is imaged by confocal microscope 4 days after an RNA injection. **(A)** The top graph shows the peak current-voltage relationships of Na_V_1.5 co-expressed with WT β1 (black) and E87Q β1 (blue), illustrating the comparable peak current amplitude. The bottom graph shows the peak current-voltage relationships of Na_V_1.5 co-expressed with WT β3 (black) and V110I β3 (blue). **(B)** The expression of Na_V_1.5 α- and β3-subunits on the *Xenopus* oocytes membrane immunostained by specific antibodies for Na_V_1.5 (green) and β3 (red).

One β1 variant, E87Q, on the extracellular domain is linked to BrS (Watanabe et al., 2008). Its co-expression with Na_V_1.5 caused a depolarizing shift in SSI curves relative to WT β1 (Figure 5A, left; Table 1). The channel recovery from inactivation was also faster than WT (Figure 5A, middle; Table 2). This faster recovery could explain the increase in channel availability. Investigation of the E87Q β1 effect on VSD-III and VSD-IV F-V curves showed no shift in half-activation voltages when compared to WT β1, but an altered slope factor of VSD-III F-V curve (*p* = 0.001) (Figure 5A, right; Table 1; Supplementary Figure S5A, right). The peak current density across multiple membrane potential was not reduced by the E87Q variant **(** Figure 6A, top), but we observed the reduction in the window current, or the area under the overlapping curves of G-V and SSI due to the shift in SSI, suggesting a loss of function in Na_V_ current.

The BrS-linked V110I variant on β3 is also found within the extracellular domain. Unlike E87Q β1, V110I β3 caused no significant shift in the G-V and SSI curves compared to WT β3 (Figure 5B, left; Table 3). The variant also did not alter the channel inactivation recovery rate, in comparison to WT β3 (WT β3: Ƭ_R_ 1 = 15.2 ± 3.8, Ƭ_R_ 2 = 1.6 ± 0.2, V110I β3: Ƭ_R_ 1 = 14.2 ± 1.2, Ƭ_R_ 2 = 1.8 ± 0.2) **(** Figure 5B, middle). Nevertheless, there was a significant reduction in the current amplitude to almost half of WT β3 across all membrane potentials **(** Figure 6A, bottom), while retaining the same peak current voltage relationship. This reduction in the peak current amplitude is consistent with decreased cell membrane expression level of Na_V_1.5 and β-subunits bearing V110I variant as illustrated by immunofluorescence staining **(** Figure 6B **)**. No significant change in the activation of VSD-IV (Supplementary Figure S5B, right), but a depolarizing shift of VSD-III activation was observed upon co-expression of V110I β3, relative to WT β3 (Figure 5B, right; Table3 **)**. None of the BrS-linked variants affected the sodium current kinetics (Supplementary Figure S5A,B, left**)**.

## Discussion

The Na_V_ β-subunits play a critical role in the regulation of cardiac Na_V_ channel function, as demonstrated in *SCN1B*- and *SCN3B*-null mice whose ventricular cardiomyocytes exhibit abnormal electrophysiology and propensity to ventricular arrhythmias (Lopez-Santiago et al., 2007; Hakim et al., 2008; Lin et al., 2015). Multiple *SCN1B* and *SCN3B* mutations are associated with cardiac arrhythmia (O’Malley and Isom, 2015; Bouza and Isom, 2018). In this study, we investigated 8 arrhythmogenic β1 or β3 variants to probe their regulation of VSD activation and its impact on Na_V_1.5 gating. The results of this investigation provide insight to the association of the β-subunits with Na_V_1.5 and mechanisms of arrhythmia.

### Structural Insights From Arrhythmogenic Non-Covalent β-Subunit Variants

The absence of β1-subunit in the cryo-EM structures of Na_V_1.5 suggests a weaker interaction with cardiac isoform than other Na_V_ homologs (Jiang et al., 2020; Li et al., 2021a; Li et al., 2021b). The substitutions of heavily glycosylated Asn-319 and Leu-316 on Na_V_1.5 potentially interfere with β1 Ig binding, and the formation of supporting hydrogen bonds (Jiang et al., 2020). Nevertheless, strong Na_V_1.5 interaction with the β3-subunit is likely conserved near VSD-III, through β3 extracellular loop (Zhu et al., 2017) and transmembrane helix (Salvage et al., 2019; Villa-Diaz et al., 2020). Such distinct interactions of β1 and β3 subunits with Na_V_1.5 results in their unique regulatory mechanisms (Zhu et al., 2017) and possible synergistic co-regulation (Ko et al., 2005; Yereddi et al., 2013).

Most disease-linked variants in this study, 3 out of 4 for each β-subunit (β1: R85H, E87Q, and D153N; β3: R6K, L10P, and V110I), were found in the extracellular domains of the β-subunits. This is not surprising given that its Ig domain resembles the Ig superfamily of cell adhesion molecules (CAMs) (Isom and Catterall, 1996) and plays a significant role in the interactions with other proteins including Na_V_ α-subunit. The extracellular domain is also necessary for the β-subunit modulatory effect on Na_V_ channel gating (Makita et al., 1996; McCormick et al., 1998; McCormick et al., 1999; Yu et al., 2005). These variants, however, do not localize to the interface of α-subunit interaction, but rather affect the conformation of the Ig loop. Since β3 Ig domains can interact and form trimers (Namadurai et al., 2014), Ig loop variants might affect the formation of the Na_V_ channel complex. Two β1 residues, R85 and E87, were proposed to be involved in the trans Ig domain interactions between two β1-subunits at the intercalated disk of adjacent cardiomyocytes, stabilizing Na_V_1.5 channels on the cell membrane in the perinexus, and maintaining the width necessary for ephaptic conduction beyond direct Na^+^ movement (Veeraraghavan et al., 2018).

One β3 variant (M161T) is found in the transmembrane segment whereas no disease-linked β1 variants have been reported in this region. This observation further supports the notion that the alpha helical transmembrane segment is critical for β3 regulation of VSD-III activation (Salvage et al., 2019) whereas β1 interaction is not dependent on this region. Instead, the β1 intracellular domain has been identified by past studies as essential for the Na_V_ channel regulation. So far, no disease-linked β3 variants have been identified within the C-terminal tail, suggesting its reduced significance with regard to the β3 regulatory mechanism. The variants in this study are not an inclusive list of all reported disease-linked variants, but it does represent the overall distribution (O’malley and Isom, 2015), providing insight into differences in β1 and β3 regulation of the Na_V_ channel.

### AF-Related Variants Reveal Unique Diseased Mechanisms of β1 and β3 Subunits

Three β1 variants (R85H, D153N and T189M) found in lone AF patients cause varying effects on the regulation of Na_V_ channel activation and inactivation gating relative to WT β1. R85H alters the slope of the VSD-III F-V curve, facilitating its activation over the range of hyperpolarized potentials where a reduction in channel availability is also observed at the same voltage range. The hyperpolarizing shift in VSD-III activation or a faster VSD-III activation at negative potentials is correlated with an enhanced fraction of Na_V_ channels undergoing closed-state inactivation (Hsu et al., 2017; Angsutararux et al., 2021) and hence the reduction in steady-state availability. The T189M variant induces a depolarizing shift in the voltage dependence of activation, while D153N β1 shows no significant difference from WT β1.

All AF-linked β1 variants, however, modify Na_V_ channel expression on the cell surface, and their corresponding recovery from inactivation. Immunofluorescence staining shows a significant increase in the membrane expression of both α- and β-subunits by D153N and T189M, that is accompanied by enhanced inactivation recovery, relative to WT. The accelerated channel recovery from inactivation can shorten the refractory period and render the cells more susceptible to irregular excitation, creating an arrhythmogenic substrate (Clancy et al., 2003).

Interestingly, a reduction in the R85H β1 surface expression does not alter the expression of the α-subunit, suggesting an altered interaction in agreement with the change in the slope of VSD-III activation and the shift in the half-maximal voltage of Q-V curve. The pattern of β1 overexpression is also distinct between D153N and T189M variants. The large clusters formed by Na_V_1.5 and T189M β1 suggest a role for the β1 intracellular domain in its association with cytoskeletal proteins (Meadow et al., 2001; McEwen et al., 2004). Past studies show that β1 facilitates Na_V_ channel trafficking to the cell surface after its assembly with the α subunit in the endoplasmic reticulum (Zimmer et al., 2002). In cardiomyocytes, phosphorylation of β1 Y181 changes the Na_V_ channel interaction with cytoskeletal proteins such as Ankyrin-G and N-Cadherin, and determines the subcellular localization (Malhotra et al., 2004). Na_V_ β1s, unlike β3, do not form trimers on their Ig domain, but through the recruitment of intracellular protein scaffold they can regulate Na_V_ channel clustering.

In contrast to the AF-related β1 variants, none of the β3 variants alter the surface expression of Na_V_1.5 nor β3-subunits relative to WT. Instead, β3 variants directly alter VSD-III activation. Two extracellular variants, R6K and L10P, enhance VSD-III contribution to pore opening, shifting the VSD-III F-V curve to positive potentials. Previous studies showed that the activation gate opening requires concerted activation of several VSDs, namely VSD-I, II and III (Chanda and Bezanilla, 2002). In Na_V_1.5, VSD-III activates at highly negative potentials (Varga et al., 2015), and thus is not rate limiting for activation gate opening. The depolarizing shift in VSD-III activation caused by β3 variants may render it the limiting transition for Na_V_ channel activation gate opening, and hence the slower sodium current activation kinetics observed in this study. The inactivation kinetics of sodium currents upon these two AF-linked β3 variant co-expression are also affected. The tightly coupled inactivation process might be decelerated by the altered channel activation kinetics. Vice versa, slowed activation kinetics could be caused by decelerated rate of inactivation (Aldrich et al., 1983). The slowed activation and inactivation kinetics can potentially cause the delayed conduction velocity and prolonged action potential duration. The alteration in the slope of VSD-III F-V curve by M161T variant implied its effect on the VSD-III activation trajectory as well.

The differential regulation of VSD-III activation has implications for their response to antiarrhythmic treatments, as past studies have shown that various antiarrhythmic drugs i.e. mexiletine, lidocaine, and ranolazine are sensitive to the VSD-III conformation. β-subunits can modulate the drug response through VSD-III regulation. Similarly, VSD-III activation is susceptible to drug binding (Moreno et al., 2016; Wang et al., 2016; Zhu et al., 2019; Zhu et al., 2021). Various modes of VSD-III modulation by these AF-linked variants may thus result in differences in drug response.

Channel recovery from inactivation of AF-linked β3 variants is also highly distinct from WT β3. Typically, the normalized current between testing and conditioning pulse monotonically increases over recovery duration to the maximum value of 1. The co-expression of AF-related variants, however, leads to recovery exceeding 1 over the short period of 10–300 ms. This phenomenon is likely a consequence of slow VSD-III deactivation which results in slow ionic current deactivation, leading to a large number of channels that remain activated upon recovery from inactivation. This feature has been previously reported when β3 is overexpressed in the excess ratio with Na_V_ α-subunit (Zhu et al., 2017). The roles of β3 in determining the distribution of Na_V_ channel clustering upon its co-assembly (Salvage et al., 2020) might also explain the resemblance of these mutational effects to that of overexpressed WT β3. The unusual channel recovery from inactivation implies that the amplitude of I_Na_ increases over the brief recovery period of high excitation frequency (>5 Hz), and hence these channels are more prone to re-opening. Additionally, the accelerated recovery may shorten the refractory period and contribute to the AF phenotype.

### Distinct Non-Covalent β-Subunit Regulations Leading to BrS Phenotypes

A rare inherited disorder, BrS, is caused primarily by the loss-of-function mutations in the *SCN5A* gene, resulting in the reduction of the peak I_Na_ amplitude. BrS-linked mutations either disrupt Na_V_ channel trafficking and decrease surface expression or alter the regulation of channel gating (Hedley et al., 2009). We found that the β3 variant, V110I, co-expression with Na_V_1.5 leads to noticeable loss in the peak I_Na_ across all membrane potential, and in the Na_V_ channel membrane expression. In contrast, the E87Q β1 variant alters the Na_V_ channel steady-state availability and recovery from inactivation.

Even though the average peak I_Na_ amplitude of E87Q β1 is not different from WT β1, we observed high variability among recordings as shown by a large standard deviation. This suggests a dynamic β1 interaction with Na_V_ α-subunit. This same variant was previously shown to reduce the peak current density and the Na_V_ channel cell surface expression in mammalian CHO cells (Baroni et al., 2017). Possibly, the amount of β1 expressed with Na_V_1.5 determines its modulation of Na_V_ channel density (Moran et al., 2000). Since the method of DNA transfection cannot precisely control the ratio of Na_V_ α- to β-subunits, the mutation effect of the peak I_Na_ density might depend on the amount of binding β1s. Multiple β-subunits can interact with the Na_V_ channel, and co-regulate the channel function. In our study, we saturate the ratio of β-to α-subunits and this might lead to the different results from past work.

### Limitations

The characterization of electrophysiological functions in this study are measured in the *Xenopus* oocytes. Unlike mammalian cells, proteins expressed in *Xenopus* oocytes do not undergo post-translational modification such as N-glycosylation, which constitutes an essential part of Na_V_ α- and β-subunits. The glycosylated channel displays modified electrophysiology (Ufret-Vincenty et al., 2001; Ednie et al., 2013). Different β-subunits can also promote distinct form of α-subunit glycosylation (Leadermann et al., 2013). Interpretation of results from our study thus does not incorporate the complexity of sugar moiety attached to the Na_V_ channel extracellular domain, and the possible mechanism of β-subunits modifying surface charges (Johnson et al., 2004; Ferrera and Moran, 2006).

Our results might not match with previous reports (Watanabe et al., 2009; Olesen et al., 2011; Ishikawa et al., 2013; Hayashi et al., 2015), due to the different experimental conditions. The ratio of β-subunits to the α-subunits, the type of cells used, and the composition of recording solution can all affect the Na_V_ channel electrophysiology, and thus complicating the comparisons when make across studies. We take advantage of the same experimental setup, to compare results across different diseased-related β variants. An extrapolation of our findings to other cell system should be done with careful consideration.

## Conclusion

Results from this study lead to three important conclusion. First, arrhythmia-linked variants on β1 and β3 subunit affect Na_V_ channel regulation through different mechanisms. This finding further supports the recent insight that β1 and β3 subunits exert differential regulation of the Na_V_ channel VSDs. Second, AF-linked variants on β3 subunit tend to directly affect its regulation on the VSD of Na_V_ α-subunit, while β1 variants show more subtle effects on channel gating, by rather modulating α-subunit or β1 expression. Lastly, AF-linked variants exhibit a wide range of effects on Na_V_ channel function, instead of commonly defined features such as decreased I_Na_ as in BrS-linked variants, suggesting diverse molecular mechanisms responsible for the arrhythmia phenotype.

## Data Availability

The data that support the findings of this study are available from the corresponding author upon request.

## Supplementary Material

Supplementary Figure S1 Voltage dependence of gating charges movements (Q-V) for all AF-linked β1 and β3 variants. Gating currents of WT-LFS channels were recorded using the same protocol as voltage dependence of channel activation (GV). The mean ± SEM is reported for groups of 3-5 cells. Left: The Q-V curves of Na_V_1.5 co-expressed with β1 variants, R85H **(A)**, D153N **(B)** or T189M **(C)**, relative to Na_V_1.5 alone and Na_V_1.5 with WT β1. Right: The Q-V curves of Na_V_1.5 co-expressed with β3 variants, R6K **(D)**, L10P **(E)** or M161T **(F)**, relative to Na_V_1.5 alone and Na_V_1.5 with WT β3.

Supplementary Figure S2 AF-linked β1 variants show no further alteration on the VSD-IV activation, in relative to WT β1. **(A)** The F-V curves of VSD-IV LFS alone (opened circle) and with WT β1 co-expression (filled circle). **(B)** The representative fluorescence traces of VSD-III (top) and VSD-IV (bottom) LFS, with or without WT β1 recorded at various voltages (−160 to 40 mV). **(C)** The F-V curves of VSD-IV LFS with AF-linked β1 variants, R85H **(left)**, D153N **(middle)** and T189M **(right)**, relative to WT β1. **(D)** The representative fluorescence traces of VSD-III LFS, co-expressed with AF-linked β1 variants, R85H **(left)**, D153N **(middle)** and T189M **(right)**, relative to WT β1. **(E)** The representative fluorescence traces of VSD-IV LFS, co-expressed with AF-linked β1 variants, R85H **(left)**, D153N **(middle)** and T189M **(right)**, relative to WT β1.

Supplementary Figure S3 AF-linked β3 variants show no alteration on the VSD-IV F-V curve, in relative to WT β1. **(A)** The representative sodium current traces recorded upon the depolarization at −20 mV for Na_V_1.5 co-expressed with WT (black) or AF-linked β3 variants, R6K (red), L10P (yellow), and M161T (blue). **(B)** The F-V curves of VSD-IV LFS alone (opened circle) and with WT β3 co-expression (filled circle). **(C)** The representative fluorescence traces of VSD-III **(top)** and VSD-IV **(bottom)** LFS co-expressed with WT β3 recorded at various voltages (−160 to 40 mV). **(D)** The F-V curves of VSD-IV LFS with AF-linked β3 variants, R6K **(left)**, L10P **(middle)** and M161T **(right)**, relative to WT β3. **(E)** The representative fluorescence traces of VSD-III LFS, co-expressed with AF-linked β3 variants, R6K **(left)**, L10P **(middle)** and M161T **(right)**, relative to WT β3. **(F)** The representative fluorescence traces of VSD-IV LFS, co-expressed with AF-linked β3 variants, R6K **(left)**, L10P **(middle)** and M161T **(right)**, relative to WT β3.

Supplementary Figure S4 Immunofluorescence staining of uninjected oocytes with Pan NaV, Myc and NaV β3. Uninjected oocytes were fixed, stained and imaged with the same settings as in Figure 4 and Figure 6. Minimal non-specific binding of these antibodies to oocytes proteins was detected.

Supplementary Figure S5 BrS-linked β variants show no alteration on the VSD-IV F-V curve, in relative to WT β1.**(A)** Left: Representative sodium current traces of NaV1.5 co-expressed with WT and E87Q β1s. Middle: The F-V curve of VSD-IV LFS co-expressed with E87Q β1, relative to WT β1. Right: Representative fluorescence traces of VSD-III and VSD-IV LFS co-expressed with E87Q β1 at various voltages (−160 to 40 mV). **(B)** Left: Representative sodium current traces of NaV1.5 co-expressed with WT and V110I β3s. Middle: The F-V curve of VSD-IV LFS co-expressed with V110I β3, relative to WT β3. Right: Representative fluorescence traces of VSD-III and VSD-IV LFS co-expressed with V110I β3 at various voltages (−160 to 40 mV).

## References

[B1] Abriel H. (2010). Cardiac Sodium Channel Na(v)1.5 and Interacting Proteins: Physiology and Pathophysiology. J. Mol. Cell Cardiol 48 (1), 2–11. 10.1016/j.yjmcc.2009.08.025 19744495

[B2] Abriel H. Kass R. S. (2005). Regulation of the Voltage-Gated Cardiac Sodium Channel Nav1.5 by Interacting Proteins. Trends Cardiovasc. Med. 15 (1), 35–40. 10.1016/j.tcm.2005.01.001 15795161

[B3] Aldrich R. W. Corey D. P. Stevens C. F. (1983). A Reinterpretation of Mammalian Sodium Channel Gating Based on Single Channel Recording. Nature 306, 436–441. 10.1038/306436a0 6316158

[B4] Angsutararux P. Kang P. W. Zhu W. Silva J. R. (2021). Conformations of Voltage-Sensing Domain III Differentially Define NaV Channel Closed- and Open-State Inactivation. J. Gen. Physiol. 153, 9. 10.1085/jgp.202112891 PMC8348240 34347027

[B5] Baroni D. Picco C. Moran O. (2017). Mutation E87Q of the β1-subunit Impairs the Maturation of the Cardiac Voltage-dependent Sodium Channel. Sci. Rep. 7, 10683. 10.1038/s41598-017-10645-y 28878239 PMC5587543

[B6] Bouza A. A. Isom L. L. (2017). Voltage-Gated Sodium Channel β Subunits and Their Related Diseases. Handb. Exp. Pharmacol. 246, 423–450. 10.1007/164_2017_48 PMC6338345 28965169

[B7] Brackenbury W. J. Isom L. L. (2011). Na Channel β Subunits: Overachievers of the Ion Channel Family. Front. Pharmacol. 2, 53. 10.3389/fphar.2011.00053 22007171 PMC3181431

[B8] Calhoun J. D. Isom L. L. (2014). The Role of Non-pore-forming β Subunits in Physiology and Pathophysiology of Voltage-Gated Sodium Channels. Handb Exp. Pharmacol. 221, 51–89. 10.1007/978-3-642-41588-3_4 24737232

[B9] Cha A. Ruben P. C. George A. L. Fujimoto E. Bezanilla F. (1999). Voltage Sensors in Domains III and IV, but Not I and II, Are Immobilized by Na+ Channel Fast Inactivation. Neuron 22, 73–87. 10.1016/S0896-6273(00)80680-7 10027291

[B10] Chanda B. Bezanilla F. (2002). Tracking Voltage-dependent Conformational Changes in Skeletal Muscle Sodium Channel during Activation. J. Gen. Physiol. 120 (5), 629–645. 10.1085/jgp.20028679 12407076 PMC2229551

[B11] Chen Q. Kirsch G. E. Zhang D. Brugada R. Brugada J. Brugada P. (1998). Genetic Basis and Molecular Mechanism for Idiopathic Ventricular Fibrillation. Nature 392 (6673), 293–296. 10.1038/32675 9521325

[B12] Clancy C. E. Tateyama M. Liu H. Wehrens X. H. Kass R. S. (2003). Non-equilibrium Gating in Cardiac Na+ Channels: an Original Mechanism of Arrhythmia. Circulation 107 (17), 2233–2237. 10.1161/01.CIR.0000069273.51375.BD 12695286

[B13] Domínguez J. N. Navarro F. Franco D. Thompson R. P. Aránega A. E. (2005). Temporal and Spatial Expression Pattern of Beta1 Sodium Channel Subunit during Heart Development. Cardiovasc. Res. 65 (4), 842–850. 10.1016/j.cardiores.2004.11.028 15721864

[B14] Ednie A. R. Horton K. K. Wu J. Bennett E. S. (2013). Expression of the Sialyltransferase, ST3Gal4, Impacts Cardiac Voltage-Gated Sodium Channel Activity, Refractory Period and Ventricular Conduction. J. Mol. Cel. Cardiol. 59, 117–127. 10.1016/j.yjmcc.2013.02.013 23471032

[B15] Ellinor P. T. Nam E. G. Shea M. A. Milan D. J. Ruskin J. N. MacRae C. A. (2008). Cardiac Sodium Channel Mutation in Atrial Fibrillation. Heart Rhythm 5 (1), 99–105. 10.1016/j.hrthm.2007.09.015 18088563

[B16] Fahmi A. I. Patel M. Stevens E. B. Fowden A. L. John J. E. III Lee K. (2001). The Sodium Channel Beta-Subunit SCN3b Modulates the Kinetics of SCN5a and Is Expressed Heterogeneously in Sheep Heart. J. Physiol. 537, 693–700. 10.1113/jphysiol.2001.01269110.1111/j.1469-7793.2001.00693.x 11744748 PMC2278985

[B17] Ferrera L. Moran O. (2006). β1-subunit Modulates the Nav1.4 Sodium Channel by Changing the Surface Charge. Exp. Brain Res. 172, 139–150. 10.1007/s00221-005-0323-4 16432696

[B18] Gaborit N. Le Bouter S. Szuts V. Varro A. Escande D. Nattel S. (2007). Regional and Tissue Specific Transcript Signatures of Ion Channel Genes in the Non-diseased Human Heart. J. Physiol. 582 (2), 675–693. 10.1113/jphysiol.2006.126714 17478540 PMC2075332

[B19] Gagnon J. A. Mowry K. L. (2011). Visualization of mRNA Localization in Xenopus Oocytes. Methods Mol. Biol. 714, 71–82. 10.1007/978-1-61779-005-8_5 21431735 PMC3181151

[B20] Glass W. G. Duncan A. L. Biggin P. C. (2020). Computational Investigation of Voltage-Gated Sodium Channel β3 Subunit Dynamics. Front. Mol. Biosci. 7, 40. 10.3389/fmolb.2020.00040 32266288 PMC7103644

[B21] Hakim P. Gurung I. S. Pedersen T. H. Thresher R. Brice N. Lawrence J. (2008). Scn3b Knockout Mice Exhibit Abnormal Ventricular Electrophysiological Properties. Prog. Biophys. Mol. Biol. 98, 251–266. 10.1016/j.pbiomolbio.2009.01.005 19351516 PMC2764399

[B22] Hayashi K. Konno T. Tada H. Tani S. Liu L. Fujino N. (2015). Functional Characterization of Rare Variants Implicated in Susceptibility to Lone Atrial Fibrillation. Circ. Arrhythm Electrophysiol. 8, 1095–1104. 10.1161/CIRCEP.114.002519 26129877

[B23] Hedley P. L. Jørgensen P. Schlamowitz S. Moolman-Smook J. Kanters J. K. Corfield V. A. (2009). The Genetic Basis of Brugada Syndrome: a Mutation Update. Hum. Mutat. 30 (9), 1256–1266. 10.1002/humu.21066 19606473

[B24] Hsu E. J. Zhu W. Schubert A. R. Voelker T. Varga Z. Silva J. R. (2017). Regulation of Na+ Channel Inactivation by the DIII and DIV Voltage-Sensing Domains. J. Gen. Physiol. 149 (3), 389–403. 10.1085/jgp.201611678 28232510 PMC5339511

[B25] Hu D. Barajas-Martinez H. Burashnikov E. Springer M. Wu Y. Varro A. (2009). A Mutation in the Beta 3 Subunit of the Cardiac Sodium Channel Associated with Brugada ECG Phenotype. Circ. Cardiovasc. Genet. 2 (3), 270–278. 10.1161/CIRCGENETICS.108.829192 20031595 PMC2801870

[B26] Hu D. Barajas-Martínez H. Medeiros-Domingo A. Crotti L. Veltmann C. Schimpf R. (2012). A Novel Rare Variant in SCN1Bb Linked to Brugada Syndrome and SIDS by Combined Modulation of Na(v)1.5 and K(v)4.3 Channel Currents. Heart Rhythm 9, 760–769. 10.1016/j.hrthm.2011.12.006 22155597 PMC3334446

[B27] Ishikawa T. Takahashi N. Ohno S. Sakurada H. Nakamura K. On Y. K. (2013). Novel SCN3B Mutation Associated with Brugada Syndrome Affects Intracellular Trafficking and Function of Nav1.5. Circ. J. 77, 959–967. 10.1253/circj.cj-12-0995 23257389

[B28] Isom L. L. Catterall W. A. (1996). Na+ Channel Subunits and Ig Domains. Nature 383 (6598), 307–308. 10.1038/383307b0 8848042

[B29] Isom L. L. De Jongh K. S. Patton D. E. Reber B. F. Offord J. Charbonneau H. (1992). Primary Structure and Functional Expression of the Beta 1 Subunit of the Rat Brain Sodium Channel. Science 256, 839–842. 10.1126/science.1375395 1375395

[B30] Isom L. L. Scheuer T. Brownstein A. B. Ragsdale D. S. Murphy B. J. Catterall W. A. (1995). Functional Co-expression of the Beta 1 and Type IIA Alpha Subunits of Sodium Channels in a Mammalian Cell Line. J. Biol. Chem. 270 (7), 3306–3312. 10.1074/jbc.270.7.3306 7852416

[B31] Jiang D. Shi H. Tonggu L. Gamal El-Din T. M. Lenaeus M. J. Zhao Y. (2020). Structure of the Cardiac Sodium Channel. Cell 180 (1), 122–e10. 10.1016/j.cell.2019.11.041 31866066 PMC6986426

[B32] Johnson D. Montpetit M. L. Stocker P. J. Bennett E. S. (2004). The Sialic Acid Component of the Beta1 Subunit Modulates Voltage-Gated Sodium Channel Function. J. Biol. Chem. 279 (43), 44303–44310. 10.1074/jbc.M408900200 15316006

[B33] Ko S.-H. Lenkowski P. W. Lee H. C. Mounsey J. P. Patel M. K. (2005). Modulation of Nav1.5 by ?1- and ?3-subunit Co-expression in Mammalian Cells. Pflugers Arch. - Eur. J. Physiol. 449, 403–412. 10.1007/s00424-004-1348-4 15455233

[B34] Laedermann C. J. Syam N. Pertin M. Decosterd I. Abriel H. (2013). β1- and β3- Voltage-Gated Sodium Channel Subunits Modulate Cell Surface Expression and Glycosylation of Nav1.7 in HEK293 Cells. Front. Cel. Neurosci. 7 (7), 137. 10.3389/fncel.2013.00137 PMC3757325 24009557

[B35] Lenkowski P. W. Shah B. S. Dinn A. E. Lee K. Patel M. K. (2003). Lidocaine Block of Neonatal Nav1.3 Is Differentially Modulated by Co-expression of Beta1 and Beta3 Subunits. Eur. J. Pharmacol. 467 (1-3), 23–30. 10.1016/s0014-2999(03)01595-4 12706451

[B36] Li R. G. Wang Q. Xu Y. J. Zhang M. Qu X. K. Liu X. (2013). Mutations of the SCN4B-Encoded Sodium Channel β4 Subunit in Familial Atrial Fibrillation. Int. J. Mol. Med. 32 (1), 144–150. 10.3892/ijmm.2013.1355 23604097

[B37] Li Z. Jin X. Wu T. Huang G. Wu K. Lei J. (2021b). Structural Basis for Pore Blockade of the Human Cardiac Sodium Channel Nav 1.5 by the Antiarrhythmic Drug Quinidine*. Angew. Chem. Int. Ed. Engl. 60 (20), 11474–11480. 10.1002/anie.202102196 33684260

[B38] Li Z. Jin X. Wu T. Zhao X. Wang W. Lei J. (2021a). Structure of Human Nav1.5 Reveals the Fast Inactivation-Related Segments as a Mutational Hotspot for the Long QT Syndrome. Proc. Natl. Acad. Sci. USA. 118 (11), e2100069118. 10.1073/pnas.2100069118 33712541 PMC7980460

[B39] Lin X. O'Malley H. Chen C. Auerbach D. Foster M. Shekhar A. (2015). Scn1b Deletion Leads to Increased Tetrodotoxin-Sensitive Sodium Current, Altered Intracellular Calcium Homeostasis and Arrhythmias in Murine Hearts. J. Physiol. 593 (6), 1389–1407. 10.1113/jphysiol.2014.277699 25772295 PMC4376420

[B40] Liu C. Tester D. J. Hou Y. Wang W. Lv G. Ackerman M. J. (2014). Is Sudden Unexplained Nocturnal Death Syndrome in Southern China a Cardiac Sodium Channel Dysfunction Disorder? Forensic Sci. Int. 236, 38–45. 10.1016/j.forsciint.2013.12.033 24529773

[B41] Lopez-Santiago L. F. Meadows L. S. Ernst S. J. Chen C. Malhotra J. D. McEwen D. P. (2007). Sodium Channel Scn1b Null Mice Exhibit Prolonged QT and RR Intervals. J. Mol. Cel. Cardiol. 43 (5), 636–647. 10.1016/j.yjmcc.2007.07.062 PMC2099572 17884088

[B42] Maier S. K. Westenbroek R. E. McCormick K. A. Curtis R. Scheuer T. Catterall W. A. (2004). Distinct Subcellular Localization of Different Sodium Channel Alpha and Beta Subunits in Single Ventricular Myocytes from Mouse Heart. Circulation 109 (11), 1421–1427. 10.1161/01.CIR.0000121421.61896.24 15007009

[B43] Makita N. Bennett P. B. George A. L. Jr. (1996). Molecular Determinants of β1Subunit-Induced Gating Modulation in Voltage-dependent Na+Channels. J. Neurosci. 16 (22), 7117–7127. 10.1523/jneurosci.16-22-07117.1996 8929421 PMC6578941

[B44] Malhotra J. D. Kazen-Gillespie K. Hortsch M. Isom L. L. (2000). Sodium Channel Beta Subunits Mediate Homophilic Cell Adhesion and Recruit Ankyrin to Points of Cell-Cell Contact. J. Biol. Chem. 275 (15), 11383–11388. 10.1074/jbc.275.15.11383 10753953

[B45] Malhotra J. D. Thyagarajan V. Chen C. Isom L. L. (2004). Tyrosine-phosphorylated and Nonphosphorylated Sodium Channel Beta1 Subunits Are Differentially Localized in Cardiac Myocytes. J. Biol. Chem. 279, 40748–40754. 10.1074/jbc.M407243200 15272007

[B46] McCormick K. A. Isom L. L. Ragsdale D. Smith D. Scheuer T. Catterall W. A. (1998). Molecular Determinants of Na+ Channel Function in the Extracellular Domain of the Beta1 Subunit. J. Biol. Chem. 273 (7), 3954–3962. 10.1074/jbc.273.7.3954 9461582

[B47] McCormick K. A. Srinivasan J. White K. Scheuer T. Catterall W. A. (1999). The Extracellular Domain of the Beta1 Subunit Is Both Necessary and Sufficient for Beta1-like Modulation of Sodium Channel Gating. J. Biol. Chem. 274 (46), 32638–32646. 10.1074/jbc.274.46.32638 10551818

[B48] McEwen D. P. Meadows L. S. Chen C. Thyagarajan V. Isom L. L. (2004). Sodium Channel Beta1 Subunit-Mediated Modulation of Nav1.2 Currents and Cell Surface Density Is Dependent on Interactions with Contactin and Ankyrin. J. Biol. Chem. 279 (16), 16044–16049. 10.1074/jbc.M400856200 14761957

[B49] Meadows L. Malhotra J. D. Stetzer A. Isom L. L. Ragsdale D. S. (2001). The Intracellular Segment of the Sodium Channel Beta 1 Subunit Is Required for its Efficient Association with the Channel Alpha Subunit. J. Neurochem. 76 (6), 1871–1878. 10.1046/j.1471-4159.2001.00192.x 11259505

[B50] Medeiros-Domingo A. Kaku T. Tester D. J. Iturralde-Torres P. Itty A. Ye B. (2007). SCN4B-encoded Sodium Channel Beta4 Subunit in Congenital Long-QT Syndrome. Circulation 116, 134–142. 10.1161/CIRCULATIONAHA.106.659086 17592081 PMC3332546

[B51] Moran O. Nizzari M. Conti F. (2000). Endogenous Expression of the beta1A Sodium Channel Subunit in HEK-293 Cells. FEBS Lett. 473 (2), 132–134. 10.1016/s0014-5793(00)01518-0 10812059

[B52] Moreno J. D. Zhu W. Mangold K. Chung W. Silva J. R. (2016). A Molecularly Detailed NaV1.5 Model Reveals a New Class I Antiarrhythmic Target. JACC Basic Transl Sci. 4 (6), 736–751. 10.1016/j.jacbts.2019.06.002 PMC6834944 31709321

[B53] Morgan K. Stevens E. B. Shah B. Cox P. J. Dixon A. K. Lee K. (2000). Beta 3: an Additional Auxiliary Subunit of the Voltage-Sensitive Sodium Channel that Modulates Channel Gating with Distinct Kinetics. Proc. Natl. Acad. Sci. U S A. 97, 2308–2313. 10.1073/pnas.030362197 10688874 PMC15797

[B54] Namadurai S. Balasuriya D. Rajappa R. Wiemhöfer M. Stott K. Klingauf J. (2014). Crystal Structure and Molecular Imaging of the Nav Channel β3 Subunit Indicates a Trimeric Assembly. J. Biol. Chem. 289 (15), 10797–10811. 10.1074/jbc.M113.527994 24567321 PMC4036194

[B55] O'Malley H. A. Isom L. L. (2015). Sodium Channel β Subunits: Emerging Targets in Channelopathies. Annu. Rev. Physiol. 77, 481–504. 10.1146/annurev-physiol-021014-071846 25668026 PMC4817109

[B56] Okata S. Yuasa S. Suzuki T. Ito S. Makita N. Yoshida T. (2016). Embryonic Type Na+ Channel β-subunit, SCN3B Masks the Disease Phenotype of Brugada Syndrome. Sci. Rep. 6, 34198. 10.1038/srep34198 27677334 PMC5039759

[B57] Olesen M. S. Jespersen T. Nielsen J. B. Liang B. Møller D. V. Hedley P. (2011). Mutations in Sodium Channel β-subunit SCN3B Are Associated with Early-Onset Lone Atrial Fibrillation. Cardiovasc. Res. 89, 786–793. 10.1093/cvr/cvq348 21051419

[B58] Olson T. M. Michels V. V. Ballew J. D. Reyna S. P. Karst M. L. Herron K. J. (2005). Sodium Channel Mutations and Susceptibility to Heart Failure and Atrial Fibrillation. JAMA 293 (4), 447–454. 10.1001/jama.293.4.447 15671429 PMC2039897

[B59] Pan X. Li Z. Huang X. Huang G. Gao S. Shen H. (2019). Molecular Basis for Pore Blockade of Human Na+ Channel Nav1.2 by the μ-conotoxin KIIIA. Science 363 (6433), 1309–1313. 10.1126/science.aaw2999 30765605

[B60] Pan X. Li Z. Zhou Q. Shen H. Wu K. Huang X. (2018). Structure of the Human Voltage-Gated Sodium Channel Nav1.4 in Complex with β1. Science 362 (6412), eaau2486. 10.1126/science.aau2486 30190309

[B61] Pan X. Li Z. Jin X. Zhao Y. Huang G. Huang X. (2021). Comparative Structural Analysis of Human Nav1.1 and Nav1.5 Reveals Mutational Hotspots for Sodium Channelopathies. Proc. Natl. Acad. Sci. USA. 118 (11), e2100066118. 10.1073/pnas.2100066118 33712547 PMC7980448

[B62] Patino G. A. Brackenbury W. J. Bao Y. Lopez-Santiago L. F. O'Malley H. A. Chen C. (2011). Voltage-gated Na+ Channel β1B: a Secreted Cell Adhesion Molecule Involved in Human Epilepsy. J. Neurosci. 31, 14577–14591. 10.1523/JNEUROSCI.0361-11.2011 21994374 PMC3212034

[B63] Riuró H. Campuzano O. Arbelo E. Iglesias A. Batlle M. Pérez-Villa F. (2014). A Missense Mutation in the Sodium Channel β1b Subunit Reveals SCN1B as a Susceptibility Gene Underlying Long QT Syndrome. Heart Rhythm 11 (7), 1202–1209. 10.1016/j.hrthm.2014.03.044 24662403

[B64] Rudokas M. W. Varga Z. Schubert A. R. Asaro A. B. Silva J. R. (2014). The Xenopus Oocyte Cut-Open Vaseline Gap Voltage-Clamp Technique with Fluorometry. J. Vis. Exp. 11 (85), 51040. 10.3791/51040 PMC4145744 24637712

[B65] Salvage S. C. Rees J. S. McStea A. Hirsch M. Wang L. Tynan C. J. (2020). Supramolecular Clustering of the Cardiac Sodium Channel Nav1.5 in HEK293F Cells, with and without the Auxiliary β3-subunit. FASEB J. 34 (3), 3537–3553. 10.1096/fj.201701473RR 31950564 PMC7079131

[B66] Salvage S. C. Zhu W. Habib Z. F. Hwang S. S. Irons J. R. Huang C. L. H. (2019). Gating Control of the Cardiac Sodium Channel Nav1.5 by its β3-subunit Involves Distinct Roles for a Transmembrane Glutamic Acid and the Extracellular Domain. J. Biol. Chem. 294 (51), 19752–19763. 10.1074/jbc.RA119.010283 31659116 PMC6926464

[B67] Sandhu A. Borne R. T. Mam C. Bunch T. J. Aleong R. G. (2017). Double Jeopardy: Long QT3 and Brugada Syndromes. Clin. Case. Rep. 5 (8), 1315–1319. 10.1002/ccr3.1064 28781849 PMC5538234

[B68] Schott J. J. Alshinawi C. Kyndt F. Probst V. Hoorntje T. M. Hulsbeek M. (1999). Cardiac Conduction Defects Associate with Mutations in SCN5A. Nat. Genet. 23 (1), 20–21. 10.1038/12618 10471492

[B69] Shen H. Liu D. Wu K. Lei J. Yan N. (2019). Structures of Human Nav1.7 Channel in Complex with Auxiliary Subunits and Animal Toxins. Science 363 (6433), 1303–1308. 10.1126/science.aaw2493 30765606

[B70] Siefani E. Bezanilla F. (1998). Cut-open Oocyte Voltage-Clamp Technique. Methods Enzymol. 293, 300–318. 9711615 10.1016/s0076-6879(98)93020-8

[B71] Tan H. L. Bink-Boelkens M. T. Bezzina C. R. Viswanathan P. C. Beaufort-Krol G. C. van Tintelen P. J. (2001). A Sodium-Channel Mutation Causes Isolated Cardiac Conduction Disease. Nature 409 (6823), 1043–1047. 10.1038/35059090 11234013

[B72] Uebachs M. Opitz T. Royeck M. Dickhof G. Horstmann M. T. Isom L. L. (2010). Efficacy Loss of the Anticonvulsant Carbamazepine in Mice Lacking Sodium Channel Beta Subunits via Paradoxical Effects on Persistent Sodium Currents. J. Neurosci. 30 (25), 8489–8501. 10.1523/JNEUROSCI.1534-10.2010 20573896 PMC6634624

[B73] Ufret-Vincenty C. A. Baro D. J. Lederer W. J. Rockman H. A. Quinones L. E. Santana L. F. (2001). Role of Sodium Channel Deglycosylation in the Genesis of Cardiac Arrhythmias in Heart Failure. J. Biol. Chem. 276 (30), 28197–28203. 10.1074/jbc.M102548200 11369778

[B74] Varga Z. Zhu W. Schubert A. R. Pardieck J. L. Krumholz A. Hsu E. J. (2015). Direct Measurement of Cardiac Na+ Channel Conformations Reveals Molecular Pathologies of Inherited Mutations. Circ. Arrhythm Electrophysiol. 8, 1228–1239. 10.1161/CIRCEP.115.003155 26283144 PMC4618166

[B75] Veeraraghavan R. Hoeker G. S. Alvarez-Laviada A. Hoagland D. Wan X. King D. R. (2018). The Adhesion Function of the Sodium Channel Beta Subunit (β1) Contributes to Cardiac Action Potential Propagation. eLife 7, e37610. 10.7554/eLife.37610 30106376 PMC6122953

[B76] Villa-Diaz F. Lopez-Nunez S. Ruiz-Castelan J. E. Salinas-Stefanon E. M. Scior T. (2020). Chemometric Models of Differential Amino Acids at the Navα and Navβ Interface of Mammalian Sodium Channel Isoforms. Molecules 25 (15), 3551. 10.3390/molecules25153551 PMC7435598 32756517

[B77] Wang H. G. Zhu W. Kanter R. J. Silva J. R. Honeywell C. Gow R. M. (2016). A Novel NaV1.5 Voltage Sensor Mutation Associated with Severe Atrial and Ventricular Arrhythmias. J. Mol. Cel. Cardiol. 92, 52–62. 10.1016/j.yjmcc.2016.01.014 PMC4789166 26801742

[B78] Wang Q. Shen J. Li Z. Timothy K. Vincent G. M. Priori S. G. (1995). Cardiac Sodium Channel Mutations in Patients with Long QT Syndrome, an Inherited Cardiac Arrhythmia. Hum. Mol. Genet. 4 (9), 1603–1607. 10.1093/hmg/4.9.1603 8541846

[B79] Watanabe H. Darbar D. Kaiser D. W. Jiramongkolchai K. Chopra S. Donahue B. S. (2009). Mutations in Sodium Channel β1- and β2-subunits Associated with Atrial Fibrillation. Circ. Arrhythm Electrophysiol. 2, 268–275. 10.1161/CIRCEP.108.779181 19808477 PMC2727725

[B80] Watanabe H. Koopmann T. T. Le Scouarnec S. Yang T. Ingram C. R. Schott J. J. (2008). Sodium Channel β1 Subunit Mutations Associated with Brugada Syndrome and Cardiac Conduction Disease in Humans. J. Clin. Invest. 118, 2260–2268. 10.1172/JCI33891 18464934 PMC2373423

[B81] Winters J. J. Isom L. L. (2016). Developmental and Regulatory Functions of Na(+) Channel Non-pore-forming β Subunits. Curr. Top. Membr. 78, 315–351. 10.1016/bs.ctm.2016.07.003 27586289

[B82] Wong H.-K. Sakurai T. Oyama F. Kaneko K. Wada K. Miyazaki H. (2005). β Subunits of Voltage-Gated Sodium Channels Are Novel Substrates of β-Site Amyloid Precursor Protein-Cleaving Enzyme (BACE1) and γ-Secretase. J. Biol. Chem. 280, 23009–23017. 10.1074/jbc.M414648200 15824102

[B83] Yereddi N. R. Cusdin F. S. Namadurai S. Packman L. C. Monie T. P. Slavny P. (2013). The Immunoglobulin Domain of the Sodium Channel β3 Subunit Contains a Surface-Localized Disulfide Bond that Is Required for Homophilic Binding. FASEB J. 27, 568–580. 10.1096/fj.12-209445 23118027 PMC3583845

[B84] Yu E. J. Ko S. H. Lenkowski P. W. Pance A. Patel M. K. Jackson A. P. (2005). Distinct Domains of the Sodium Channel Beta3-Subunit Modulate Channel-Gating Kinetics and Subcellular Location. Biochem. J. 392 (3), 519–526. 10.1042/BJ20050518 16080781 PMC1316291

[B85] Yu F. H. Westenbroek R. E. Silos-Santiago I. McCormick K. A. Lawson D. Ge P. (2003). Sodium Channel Beta4, a New Disulfide-Linked Auxiliary Subunit with Similarity to Beta2. J. Neurosci. 23 (20), 7577–7585. 10.1523/jneurosci.23-20-07577.2003 12930796 PMC6740763

[B86] Yuan L. Koivumäki J. T. Liang B. Lorentzen L. G. Tang C. Andersen M. N. (2014). Investigations of the Navβ1b Sodium Channel Subunit in Human Ventricle; Functional Characterization of the H162P Brugada Syndrome Mutant. Am. J. Physiol. Heart Circ. Physiol. 306, H1204–H1212. 10.1152/ajpheart.00405.2013 24561865

[B87] Zhu W. Mazzanti A. Voelker T. L. Hou P. Moreno J. D. Angsutararux P. (2019). Predicting Patient Response to the Antiarrhythmic Mexiletine Based on Genetic Variation. Circ. Res. 124 (4), 539–552. 10.1161/CIRCRESAHA.118.314050 30566038 PMC6588292

[B88] Zhu W. Voelker T. L. Varga Z. Schubert A. R. Nerbonne J. M. Silva J. R. (2017). Mechanisms of Noncovalent β Subunit Regulation of NaV Channel Gating. J. Gen. Physiol. 149 (8), 813–831. 10.1085/jgp.201711802 28720590 PMC5560778

[B89] Zhu W. Wang W. Angsutararux P. Mellor R. L. Isom L. L. Nerbonne J. M. (2021). Modulation of the Effects of Class Ib Antiarrhythmics on Cardiac NaV1.5-encoded Channels by Accessory NaVβ Subunits. JCI Insight 6, 143092. 10.1172/jci.insight.143092 34156986 PMC8410097

[B90] Zimmer T. Biskup C. Bollensdorff C. Benndorf K. (2002). The B1 Subunit but Not the B2 Subunit Colocalizes with the Human Heart Na+ Channel (hH1) Already within the Endoplasmic Reticulum. J. Membr. Biol. 186, 13–21. 10.1007/s00232-001-0131-0 11891585

