# Supplementary figures and images for: Molecular Pathology of Sodium Channel Beta-Subunit Variants
Source: Front Pharmacol. 2021 Nov 19;12:761275. doi: 10.3389/fphar.2021.761275 (PMC8640220; doi:10.3389/fphar.2021.761275)

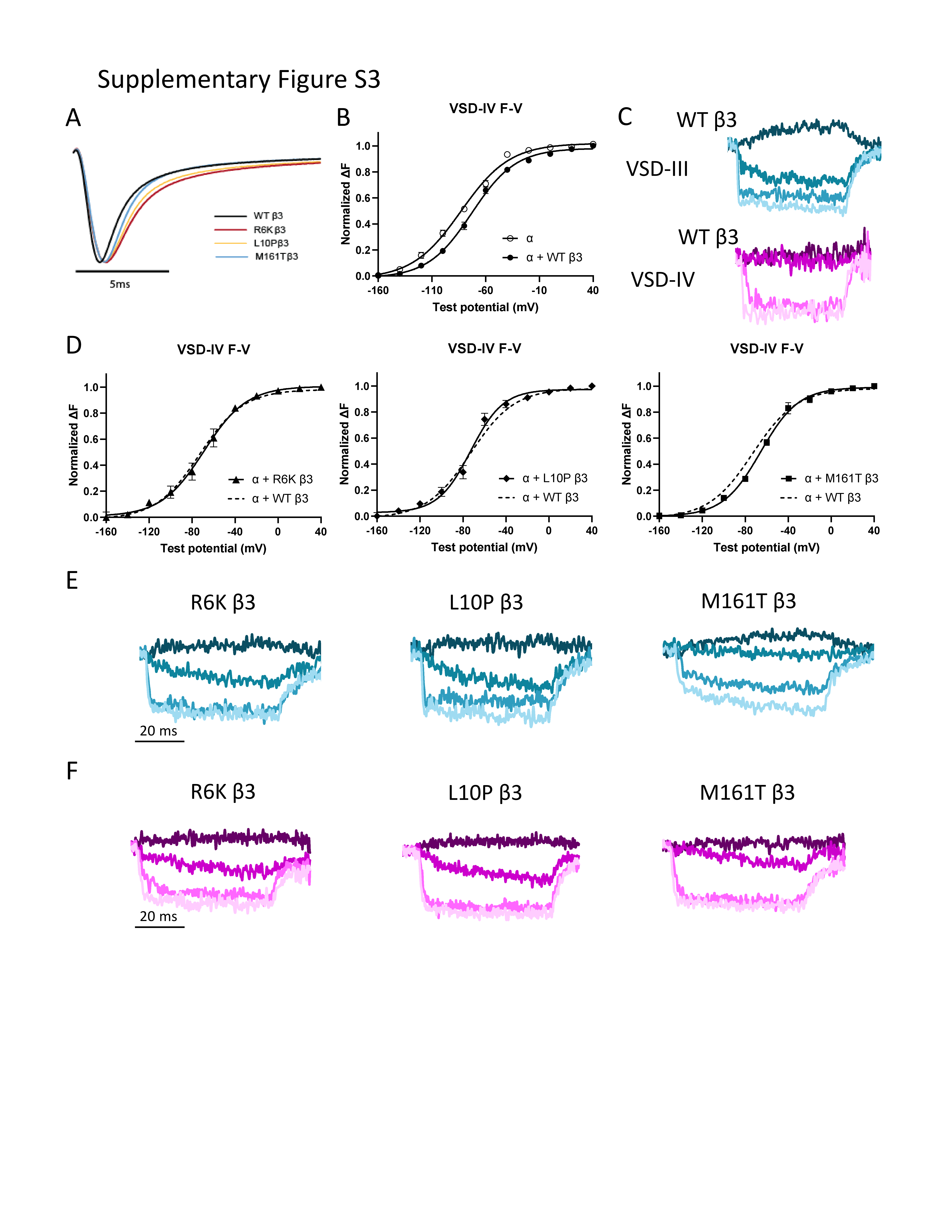

Supplement: Supplementary file 1 [file Image3.TIF]

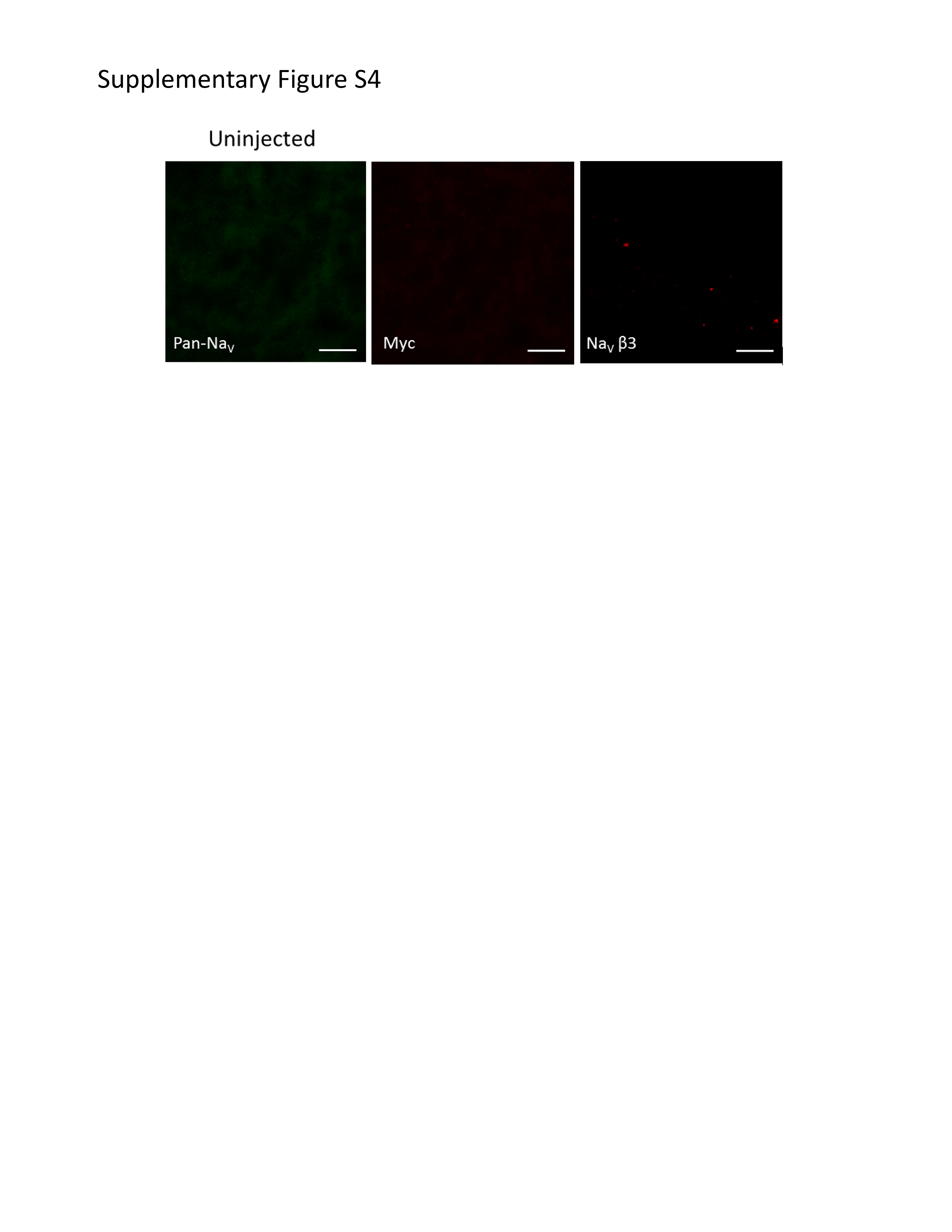

Supplement: Supplementary file 2 [file Image4.TIF]

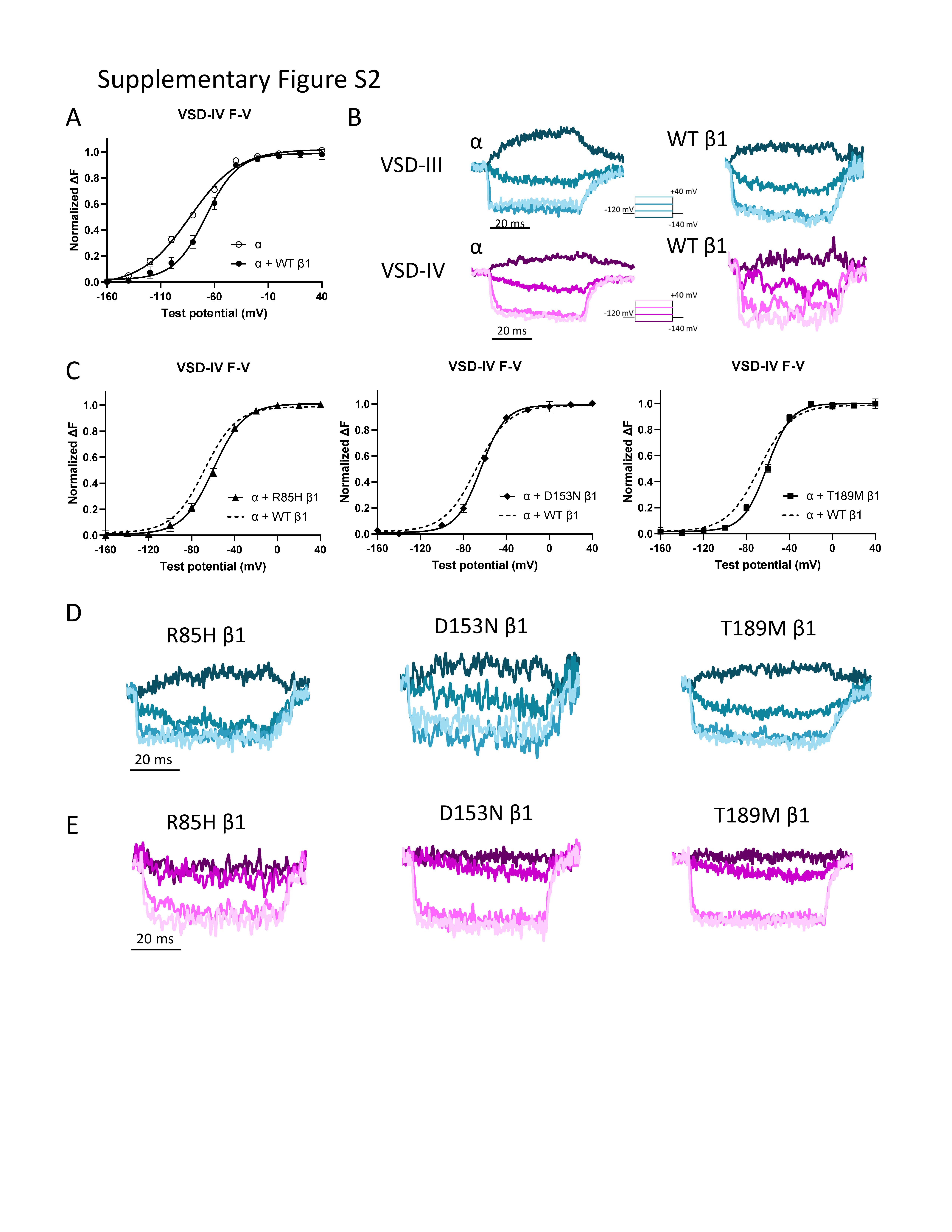

Supplement: Supplementary file 3 [file Image2.TIF]

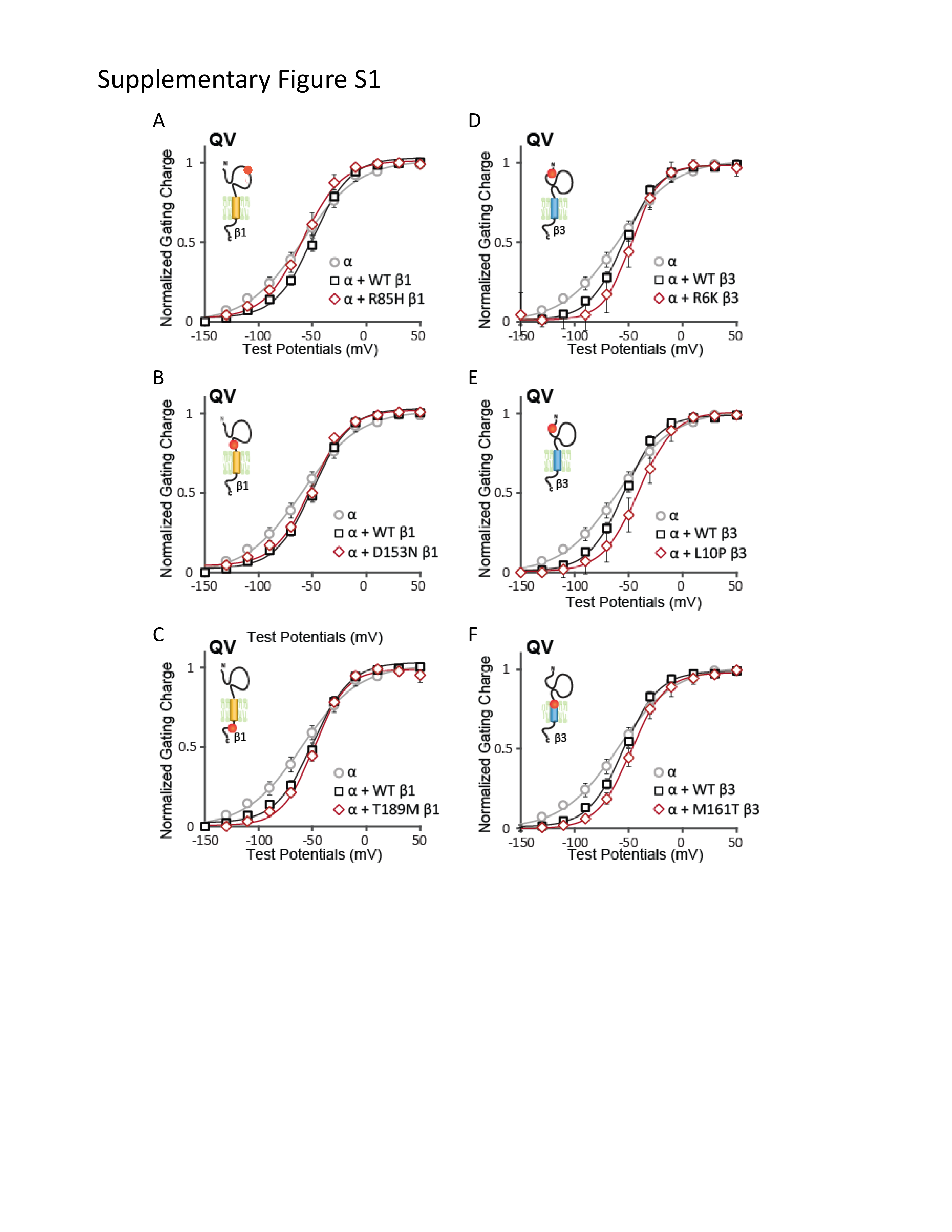

Supplement: Supplementary file 4 [file Image1.TIF]

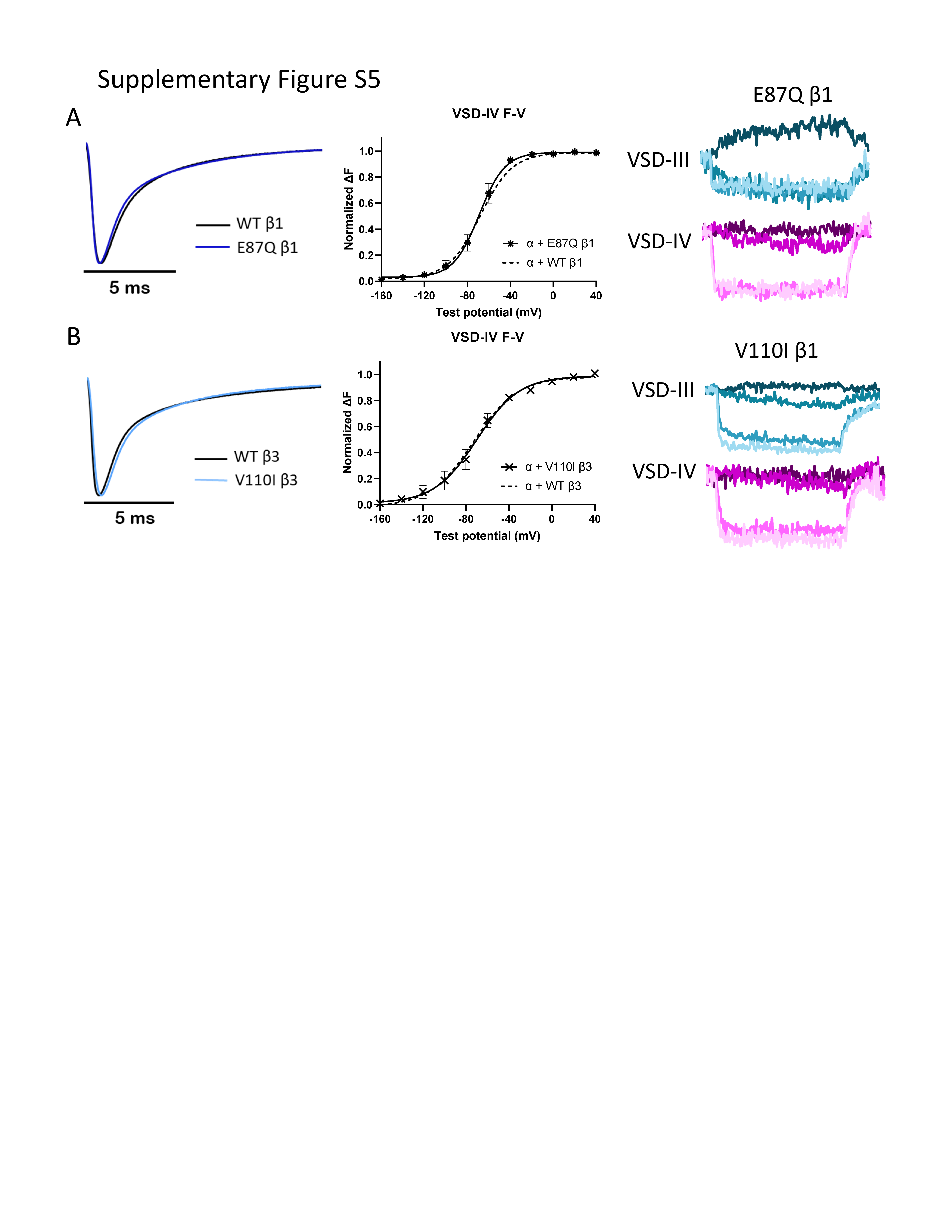

Supplement: Supplementary file 5 [file Image5.TIF]
