# Supplementary material for: Molecular Pathology of Sodium Channel Beta-Subunit Variants
Source: Front Pharmacol. 2021 Nov 19;12:761275. doi: 10.3389/fphar.2021.761275 (PMC8640220; doi:10.3389/fphar.2021.761275)
Supplement: Supplementary file 6 [file DataSheet1.docx]

**Supplementary Table S1: Summary of the parameters (V_1/2_ and k) fitted to Boltzmann equation for Na_V_1.5 α-subunit alone or with β1 and β3 subunits. P-values are computed from one-way ANOVA followed by Donnett’s post hoc test relative to the control group of α-subunit alone.**

|  |  | **WT α** | **WT α + β1** | **WT α + β3** |
| --- | --- | --- | --- | --- |
| **G-V** |  |  |  |  |
|  | **V_1/2_** | -35.8 ± 1.4 | -37.6 ± 0.7 | -31.9 ± 0.3 |
|  | **p-value** |  | 0.30 | 0.09 |
|  | **k [n]** | 6.8 ± 0.2 [4] | 8.8 ± 0.4 [5] | 7.2 ± 0.2 [4] |
| **SSI** |  |  |  |  |
|  | **V_1/2_** | -81.4 ± 2.3 | -71.4 ± 1.6 | -72.4 ± 1.5 |
|  | **p-value** |  | 0.011 (*) | 0.032 (*) |
|  | **k** **[n]** | 9.9 ± 0.7 [6] | -6.0±0.1 [4] | -8.2±0.3 [3] |
| **DIII F-V** |  |  |  |  |
|  | **V_1/2_** | -120.7 ± 4.8 | -134.6 ± 7.1 | -88.0 ± 3.2 |
|  | **p-value** |  | 0.14 | <0.001 (***) |
|  | **k [n]** | 25.6 ± 0.7 [4] | 31.3 ± 0.5 [4] | 24.0 ± 2.0 [7] |
| **DIV F-V** |  |  |  |  |
|  | **V_1/2_** | -83.2 ± 1.7 | -67.9 ± 3.1 | -73.7 ± 1.6 |
|  | **p-value** |  | 0.001 (***) | 0.02 (*) |
|  | **k [n]** | 23.0 ± 2.0 [5] | 15.1 ± 0.4 [4] | 20.5 ± 1.2 [4] |
|  |  |  |  |  |

**Supplementary Table S2: Summary of Q-V relationship fits for Na_V_1.5 α- and β-subunits, reported as mean values ± SEM. P-values of each β variants compared to WT were computed from One-way ANOVA followed by Donnett’s post hoc test.**

|  | **k** | **n** | **V_1/2_ (mV)** | **ΔV_1/2_** | **p-value** |
| --- | --- | --- | --- | --- | --- |
| **WT-LFS α** | 25.9 ± 2.8 | 4 | -60.7 ± 6.9 |  |  |
|  |  |  |  |  |  |
| **WT-LFS α + WT β1** | 17.3 ± 1.2 | 4 | -49.8 ± 2.1 |  |  |
| **WT-LFS α + R85H β1** | 17.7 ± 0.7 | 3 | -58.7 ± 2.1 | -8.9 | 0.03 (*) |
| **WT-LFS α + D153N β1** | 17.4 ± 1.2 | 3 | -51.1 ± 1.3 | -1.3 | 0.71 |
| **WT-LFS α + T189M β1** | 15.0 ± 1.5 | 3 | -48.1 ± 3.5 | 1.7 | 0.05 |
|  |  |  |  |  |  |
| **WT-LFS α + WT β3** | 17.3 ± 2.5 | 4 | -55.1 ± 2.3 |  |  |
| **WT-LFS α + R6K β3** | 13.5 ± 5.3 | 3 | -45.1 ± 3.5 | 10.0 | 0.05 (*) |
| **WT-LFS α + L10P β3** | 16.6 ± 1.2 | 3 | -41.4 ± 3.6 | 13.7 | 0.02 (*) |
| **WT-LFS α + M161T β3** | 16.5 ± 3.4 | 4 | -47.0 ± 2.6 | 8.1 | 0.05 (*) |
|  |  |  |  |  |  |
